# Elucidating prognostic significance of purine metabolism in colorectal cancer through integrating data from transcriptomic, immunohistochemical, and single‐cell RNA sequencing analysis

**DOI:** 10.1002/1878-0261.70010

**Published:** 2025-02-27

**Authors:** Sungyeon Kim, Myunghee Kang, Soyeon Jeong, Jisup Kim, Kyoung Oh Kim, Won‐Suk Lee, Jeong‐Heum Baek, Jung Ho Kim, Seungyoon Nam

**Affiliations:** ^1^ Department of Genome Medicine and Science, Gachon Institute of Genome Medicine and Science, Gachon University Gil Medical Center, Gachon University College of Medicine Gachon University Incheon Korea; ^2^ Department of Pathology, Gachon University Gil Medical Center, Gachon University College of Medicine Gachon University Incheon Korea; ^3^ Department of Internal Medicine, Gachon University Gil Medical Center, Gachon University College of Medicine Gachon University Incheon Korea; ^4^ Department of Surgery, Gachon University Gil Medical Center, Gachon University College of Medicine Gachon University Incheon Korea; ^5^ Gachon Medical Research Institute, Gachon Biomedical Convergence Institute Gachon University Gil Medical Center Incheon Korea; ^6^ Department of Translational‐Clinical Medicine, Gachon Advanced Institute for Health Sciences and Technology (GAIHST) Gachon University Incheon Korea; ^7^ Department of Health Sciences and Technology, Gachon Advanced Institute for Health Sciences and Technology (GAIHST) Gachon University Incheon Korea

**Keywords:** colorectal cancer, immunohistochemistry, purine metabolism, survival

## Abstract

Colorectal cancer (CRC) is widely recognized for its high prevalence and significant mortality rates, and purine metabolism has been serving as a potential therapeutic target. However, purine metabolism has not yet been validated as a prognostic marker through immunohistochemical analysis. In this study, we utilized a combination of bulk transcriptome analysis, immunohistochemistry (IHC), and single‐cell RNA sequencing (scRNA‐seq) to assess the clinical relevance of purine metabolism in CRC. Low expression levels of five purine metabolism‐related genes—*ADSL*, *APRT*, *ADCY3*, *NME3*, and *NME6*—were associated with worse prognosis in CRC patient subgroups, including wild‐type *TP53*, mutant *TP53*, and microsatellite‐stable phenotypes. IHC‐based validation showed that NME3 expression was an independent prognostic factor, whereas ADSL and NME6 expressions were associated with clinical variables in prediction of prognosis. Notably, NME3 expression predicted a high risk in patients with early‐stage CRC, while ADSL and NME6 expressions were predictive in late‐stage CRC. scRNA‐seq analysis showed that four genes, excluding *NME6*, had low expression levels in epithelial cells at the late‐stage CRC. Despite the reversible nature of purine metabolism reactions, we demonstrated a consistent directional expression of these five prognostic purine metabolism‐related proteins in CRC tissues. We suggest that alterations in purine metabolism could serve as a clinically useful prognostic marker in CRC.

## Graphical abstract

Low expression of five purine metabolism‐related genes (*ADSL*, *APRT*, *ADCY3*, *NME3*, *NME6*) was correlated with poor survival in colorectal cancer. Immunohistochemistry analysis showed that low NME3 (early stage) and low ADSL/NME6 (late stage) levels were associated with high risk. scRNA‐seq revealed lower expression of *ADSL*, *APRT*, *ADCY3*, and *NME3* in epithelial cells of late‐stage patients.

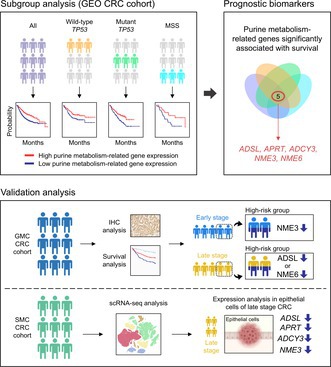

**Abbreviations**
ADCY3: adenylate cyclase 3
ADSL: adenylosuccinate lyase
AMP: adenosine monophosphate
AMPK: AMP‐activated protein kinase
APRT: adenine phosphoribosyltransferase
AUC: area under the ROC curve
cAMP: cyclic AMP
CI: confidence interval
CPM: counts per million
CRC: colorectal cancer
DFS: disease‐free survival
DSS: disease‐specific survival
GEO: Gene Expression Omnibus
GMC: Gil Medical Center
HR: hazard ratio
IHC: immunohistochemistry
KEGG: Kyoto Encyclopedia of Genes and Genomes
MSI: microsatellite instability
MSI‐H: MSI‐high
MSS: microsatellite stable
NME3: nucleoside diphosphate kinase 3
NME6: nucleoside diphosphate kinase 6
OR: odds ratio
OS: overall survival
ROC: receiver operating characteristic
scRNA‐seq: single‐cell RNA sequencing
SMC: Samsung Medical Center
TMA: tissue microarray
t‐SNE: t‐distributed stochastic neighbor embedding

## 1 Introduction

Colorectal cancer (CRC), a prevalent and potentially fatal cancer affecting the colon or rectum, represents a significant global health issue [1, 2]. Despite advances in medical treatment, CRC remains the second leading cause of cancer‐related deaths in 2020 [2]. To improve patient survival rates, it is crucial to develop diverse biomarkers with various targets for clinical application [3].

Identifying clinically validated biomarkers specific to patient subgroups is essential for advancing personalized prevention and treatment strategies for CRC [4]. Additionally, biomarkers and substances present in body fluids or tissues show promise for enhancing treatment efficacy and safety [5]. Prognostic biomarkers, in particular, are critical for assessing the potential outcomes of disease remission [5]. Continued research on prognostic biomarkers and strategies to establish their clinical utility is vital for improving overall outcomes in patients with CRC [5].

Purine metabolism is essential for DNA and RNA synthesis, cellular energy production, and signaling pathways through the generation of adenosine triphosphate and guanosine triphosphate [6]. When cellular purine levels become depleted, the formation of a complex known as the ‘purinosome’ activates purine metabolism [6]. Dysregulation of purine metabolism is linked to various diseases, including cancer [7]. In CRC, purine metabolism is associated with tumorigenesis [8] and is notably disrupted in CRC patients compared with healthy individuals [9]. Purine metabolism generates purine nucleotides that promote cancer growth and proliferation, making it a target for cancer therapy strategies [10]. However, metabolic intermediates such as adenosine monophosphate (AMP) and 5‐aminoimidazole‐4‐carboxamide ribonucleotide can activate AMP‐activated protein kinase (AMPK) and suppress tumor progression [11]. Thus, while purine metabolism generally supports tumor progression, specific genes within this pathway may have tumor‐suppressive roles [12]. This dual role highlights the complexity of purine metabolism in cancer biology.

While purine metabolism is widely recognized as a therapeutic target in cancer, its potential as a prognostic biomarker has not been thoroughly investigated, particularly in CRC [13, 14, 15]. Moreover, it has not been validated as a prognostic marker in CRC using methods other than immunohistochemistry (IHC). Although an analysis of overall survival (OS) based on adenylosuccinate lyase (ADSL) expression was reported in a CRC cohort, the findings were not statistically significant [16]. Previous studies have primarily focused on purine metabolism's role in tumor progression and drug resistance [17, 18]. However, there is a lack of comprehensive research validating purine metabolism‐related genes or proteins as prognostic markers using clinical specimens and survival analyses, especially through cost‐effective and clinically implementable methods such as IHC [19, 20]. Furthermore, the expression patterns and prognostic significance of purine metabolism‐related genes in key CRC subgroups (e.g., *TP53* mutation, microsatellite instability [MSI] status) remain underexplored.

To address this gap, we combined transcriptomic analysis, IHC validation, and single‐cell RNA sequencing to systematically evaluate the prognostic potential of purine metabolism‐related genes in CRC. Our study first explored the prognostic and clinical relevance of gene expression related to purine metabolism across multiple publicly available CRC patient datasets. We focused on clinicopathological features associated with better OS because OS serves as a critical endpoint in evaluating the long‐term impact of prognostic biomarkers. OS reflects the comprehensive outcome of CRC progression and treatment efficacy, making it a highly relevant criterion for translational studies. These findings were subsequently validated using IHC and survival analyses in an independent cohort. We also investigated whether purine metabolism proteins correlated with clinical variables or served as independent prognostic markers within the same cohort. Lastly, single‐cell transcriptomic analysis of another independent cohort was conducted to examine the relationship between genes associated with purine metabolism and tumor progression.

## 2 Materials and methods

### 2.1 CRC transcriptome data collection and preprocessing

To identify patients with CRC with available survival data, we searched transcriptome datasets using the keywords ‘colorectal cancer’ and ‘survival’ in the Gene Expression Omnibus (GEO) database [21]. A total of 16 datasets (GSE161158, GSE72969, GSE72968, GSE38832, GSE39084, GSE29621, GSE39582, GSE30378, GSE31595, GSE24550, GSE24549, GSE17537, GSE17536, GSE16125, GSE12945, and GSE106535) were retrieved as of July 9, 2021 (Table S1) [22, 23, 24, 25, 26, 27, 28, 29, 30, 31, 32, 33]. This resulted in data from 1931 patients, of which 1147 had available OS information. Subsequent analyses focused on this subset, referred to as ‘the GEO cohort.’ The clinical characteristics of the GEO cohort, including age, sex, TNM stage, OS information, and country of origin, were also collected (Table S1). The inclusion criteria for dataset selection required that patients had OS data available. The exclusion criteria involved removing datasets explicitly labeled as containing survival information but lacking actual OS data. As a result, the following datasets were excluded: GSE161158, GSE38832, GSE30378, GSE31595, GSE24550, GSE24549, and GSE106535.

The datasets were processed in r (version 4.1.0) using the robust multichip average algorithm from the ‘oligo’ package [34]. To correct for batch effects, the ComBat algorithm from the ‘sva’ package was applied [35]. The ComBat algorithm, leveraging an empirical Bayesian approach, addresses batch effects in high‐dimensional datasets [35]. It achieves precise adjustment by modeling batch factors independently of biological variables [35]. Gene annotation was performed using the Bioconductor ‘annotation’ package [36].

### 2.2 Survival analysis according to gene expression in the GEO cohort

A total of 115 purine metabolism‐related genes were sourced from the Molecular Signature Database gene sets [37]. To explore the relationship between gene expression and OS, we conducted a survival analysis. For survival analysis for all patients, the patients were divided into high and low gene expression groups. To classify patients into low‐ and high‐expression groups in the all‐patients analysis, we used the optimal cutoff values determined using the *surv_cutpoint* function from the ‘survminer’ r package (version 4.1.0) (Table S2). For patient subgroups (e.g., wild‐type *TP53*, mutant *TP53*, microsatellite stable [MSS]), the median expression levels were used as cutoff values to define low‐ and high‐expression groups (Table S3). Subsequently, patients with gene expression values equal to or above the cutoff were classified into the high‐expression group, while those with values below the cutoff were classified into the low‐expression group. Kaplan–Meier plots were generated using the ‘survminer’ package in r.

### 2.3 Patient sample collection

This samples were collected between April 2010 and January 2013 on patients who underwent CRC surgery at Gil Medical Center (GMC). Inclusion criteria required that patients had primary CRC, underwent surgery, and had preserved tumor pathology blocks. A total of 590 patients were included in this analysis. Exclusion criteria were recurrent CRC, altered normal bowel structure from previous surgery, presurgery chemotherapy or radiation therapy, and treatment for other cancers prior to CRC surgery. Epidemiological data were collected from the patients.

All study protocols and procedures were approved by the Institutional Review Board of GMC (GBIRB2016‐318). Prior to sample collection, all participants were informed about the study and provided written consent. The study adhered to the Declaration of Helsinki and the Code of Ethics of the World Medical Association.

### 2.4 Tissue microarray (TMA) and IHC

After microdissecting the paraffin blocks, we performed hematoxylin and eosin staining and reviewed the pathological findings. Two tumor cores were marked on the corresponding paraffin blocks. Using a tissue microarray machine, cylindrical tumor tissues with a diameter of 2 mm were extracted and transferred to new paraffin blocks. Each cylindrical tissue sample from the 69 patients was embedded in a paraffin block to create a new TMA. Specifically, each TMA consisted of 69 tissue cores, and a total of nine TMAs were constructed, yielding 621 tissue cores. Patients with noncolorectal cancer or those who had received preoperative chemotherapy or radiotherapy were excluded, leaving 590 patients for analysis. The TMA block was then cut to a thickness of 4 μm using a microtome, mounted on slides in a specific orientation, and dried.

TMA staining was conducted as described in our previous study [19]. TMA slides were baked, deparaffinized, and rehydrated to inhibit endogenous peroxidase activity, followed by antigen retrieval. Samples were preincubated for 30 min in 10% normal goat serum (Catalog #31872, Invitrogen, Danvers, MA, USA) to prevent nonspecific staining and then incubated overnight at 4 °C with specific antibodies in a humidified container. Tissue slides were processed using a nonbiotin horseradish peroxidase detection system following the manufacturer's instructions (Leica Bond‐III system, GmbH, Nussloch, Germany). The antibodies used included: anti‐ADSL antibody (catalog # HPA000525, rabbit polyclonal, 1 : 100, Atlas Antibodies, Stockholm, Sweden), anti‐adenine phosphoribosyltransferase (APRT) antibody (catalog # HPA026681, rabbit polyclonal, 1 : 100, Atlas Antibodies), anti‐adenylate cyclase 3 (ADCY3) antibody (catalog # PAC‐301AP, rabbit polyclonal, 1 : 500, Thermo Fisher Scientific, Waltham, MA, USA), anti‐nucleoside diphosphate kinase 3 (NME3) antibody (catalog # ab181257, rabbit monoclonal, 1 : 100, Abcam, Cambridge, UK), and anti‐nucleoside diphosphate kinase 6 (NME6) antibody (catalog # HPA017909, rabbit polyclonal, 1 : 200, Atlas Antibodies).

TMA staining was conducted as described in our previous study [19]. The TMA slides were independently reviewed twice by two experienced pathologists (MK and JK), without any additional information to prevent bias. The scoring system was as follows: score 0, no staining; score 1+, faint or barely discernible cytoplasmic staining in any tumor cell; score 2+, moderately smooth granular cytoplasmic staining; and score 3+, strong and diffuse cytoplasmic staining. ADCY3 was categorized as either positive or negative.

### 2.5 Survival analysis based on protein expression and clinical information

To assess the clinical significance of the candidate biomarkers (i.e., ADSL, APRT, ADCY3, NME3, and NME6), we performed correlation analyses between protein expression and clinical characteristics. We also evaluated whether these biomarkers were prognostic factors for survival, which is a crucial oncological outcome.

We categorized patients based on their IHC scores into two groups: those with scores of 0 or 1+ were classified into the low protein expression group, and those with scores of 2+ or 3+ were classified into the high protein expression group. Patients with CRC were further stratified by TNM stage into early stage (I or II) and late stage (III or IV). The early‐ and late‐stage groups were subdivided based on IHC scores into four subgroups: high expression and early stage, low expression and early stage, high expression and late stage, and low expression and late stage. Kaplan–Meier survival plots were generated using the same methodology as in previous survival analyses.

### 2.6 Analysis of association between protein expression and clinical variables

Chi‐squared tests and logistic regression analyses were employed to explore the relationship between protein expression and clinical variables (e.g., sex, age, smoking, family history, diabetes diagnosis, anemia, cancer differentiation, T stage, lymph node metastasis, and distant metastasis). Initially, chi‐squared tests identified clinical variables significantly associated with protein expression. Subsequently, multivariate logistic regression was performed to determine the independent variables associated with protein expression among the identified clinical variables. All statistical analyses were conducted using IBM spss Statistics for Windows, version 22.0 (IBM Corp., Armonk, NY, USA).

### 2.7 Single‐cell RNA sequencing (scRNA‐seq) data analysis

We searched scRNA‐seq datasets using the keywords ‘colorectal cancer,’ ‘single cell,’ and ‘RNA sequencing’ in GEO database [21]. The inclusion criteria for dataset selection required a sample size of 20 or more Korean patients with colorectal cancer. The exclusion criteria were datasets involving colorectal cancer patients who were not Korean. To analyze the scRNA‐seq data for patients with CRC, we utilized the GSE132465 dataset from GEO [38]. This dataset comprised 63 689 cells from 23 primary CRC patients and 10 normal mucosa samples from Korean CRC patients at the Samsung Medical Center (SMC). Analysis focused on 23 primary CRC samples, containing 47 282 cells. The raw count matrix was processed using the ‘Seurat’ package [39] in r. Counts per million (CPM) were calculated from the count matrix and log2‐transformed after adding one to each CPM value. For clustering, 2000 genes with high expression levels were selected as variable features using the *FindVariableGenes* function. Visualization was achieved using t‐distributed stochastic neighbor embedding (t‐SNE).

To evaluate whether the expression levels of a specific gene differed between the early‐stage and late‐stage groups, the medians of the two groups were compared. The statistical significance of gene expression difference between the two groups was assessed using two‐sample *t*‐tests, with a *P* value cutoff 0.05.

### 2.8 Gene and protein expression correlation analysis

Pearson's correlation analysis was conducted to assess the correlation between gene and protein expression levels. Gene expression correlation analysis involved 1147 samples and 17 469 epithelial cells from 23 patients with CRC in the GEO and SMC cohorts. Protein expression correlation was analyzed using IHC scores from 590 CRC patients in the GMC cohort. A correlation matrix was generated and visualized as a heatmap using the ‘corrplot’ package.

### 2.9 Time‐dependent receiver operating characteristic (ROC) analysis

We performed time‐dependent ROC analysis using the ‘survivalROC’ package [40]. In the GEO cohort, we conducted ROC analyses to evaluate the prediction of 1‐, 3‐, and 5‐year OS outcomes in all patients, as well as in the subgroups of wild‐type *TP53*, mutant *TP53*, and MSS. Similarly, in the GMC cohort, we assessed the prediction of 1‐, 3‐, and 5‐year disease‐free survival (DFS) and disease‐specific survival (DSS) outcomes in all patients and the same subgroups (wild‐type *TP53*, mutant *TP53*, and MSS). Furthermore, we performed ROC analyses in the GMC cohort to evaluate the prediction of 1‐, 3‐, and 5‐year DFS and DSS outcomes in early‐ and late‐stage subgroups.

### 2.10 Statistical analysis

Survival rate differences between patient groups were assessed using the log‐rank test. The chi‐squared test was employed for categorical variable analysis. Binary logistic regression analysis estimated the odds ratio (OR) and its 95% confidence interval (CI) for each clinical variable related to protein expression in patients with CRC. Multivariate survival analysis was performed using the Cox proportional hazards model, with hazard ratio (HR) and 95% CI calculated. Statistical significance was determined at a threshold of *P* < 0.05.

## 3 Results

### 3.1 Overview of our study using multiple CRC cohorts

**Fig. 1:**
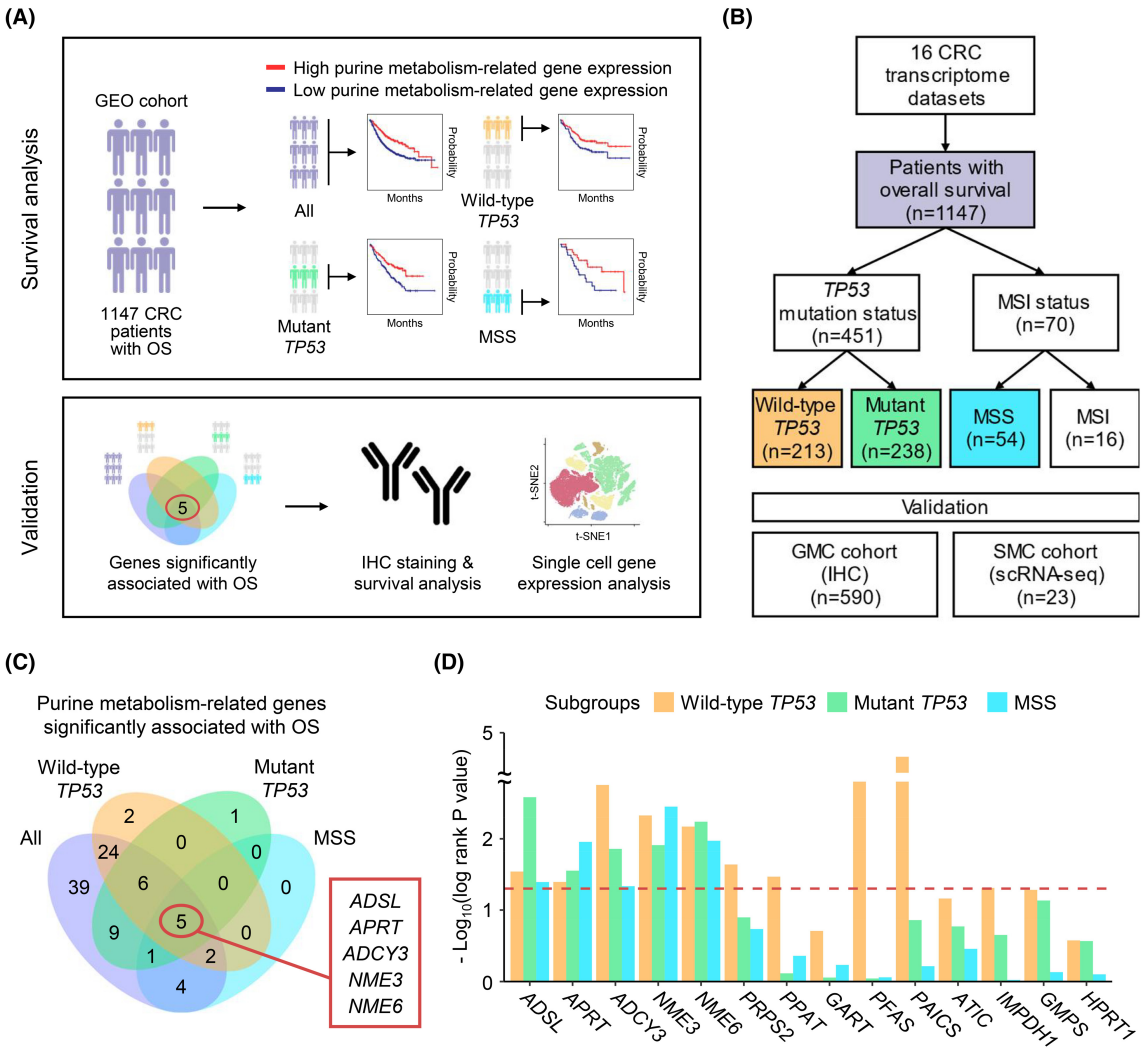
Identification of five prognostic purine metabolism‐related genes from multiple public CRC datasets. (A) Graphical overview of this study. (B) Overview of patient datasets used for analysis. CRC patients with OS were classified into four subgroups based on *TP53* mutation and MSI status. (C) Venn diagram of significant (log‐rank *P* < 0.05) survival‐related genes identified in each of the four CRC patient groups. (D) The bar plot shows the −log10 transformed log‐rank *P* values of the five prognostic purine metabolism‐related genes and other purine metabolism‐related genes associated with CRC in each CRC subgroup. Log‐rank tests were used for OS analysis. CRC, colorectal cancer; MSI, microsatellite instability; OS, overall survival.

We have provided a graphical overview to illustrate the overall process of identifying purine metabolism‐related genes associated with survival in patients with CRC (Fig. 1A). In this study, we emphasized the significance of the *TP53* gene in CRC, given its crucial role in the disease due to *TP53* mutations [41]. Therefore, we categorized patients into two subgroups based on the presence or absence of *TP53* mutations. Additionally, considering the clinical aspect, the MSI status is important [42], we distinguished between MSS and MSI subgroups. The treatment varies according to MSI status [43]. Chemotherapy is preferred for MSS patients, while immunotherapy is preferred for MSI‐high (MSI‐H) patients [43]. Additionally, adjuvant chemotherapy may differ among stage II patients [43]. Adjuvant chemotherapy is not recommended for MSI‐H colorectal cancer but is recommended for MSS colorectal cancer [43]. Therefore, it is important to classify patients using MSI status. Our goal was to explore potential common biomarkers across all patients and within the four distinct subgroups: wild‐type *TP53* (*n* = 213), mutant *TP53* (*n* = 238), MSS (*n* = 54), and MSI (*n* = 16). This approach aimed to identify shared genetic elements applicable to heterogeneous CRC populations (Fig. 1B).

Furthermore, after identifying potential biomarker candidates, we assessed their clinical significance by examining clinicopathological characteristics. To validate our findings, we conducted clinical validation focusing on survival outcomes. This comprehensive approach allowed us to identify common biomarker candidates across subgroups, evaluate their clinical significance, and validate their impact on survival, thus providing a robust foundation for prognostic biomarkers applicable to all patients with CRC.

Notably, in the survival analysis of the MSI subgroup, no significant genes were identified due to a limited sample size. However, in the wild‐type *TP53*, mutant *TP53*, and MSS subgroups, we identified five common genes (*ADSL*, *APRT*, *ADCY3*, *NME3*, and *NME6*) with statistically significant differences (log‐rank *P* < 0.05) in survival rates between high‐ and low‐expression groups (red circle in Fig. 1C). Other purine metabolism‐related genes associated with CRC [44, 45] were not significantly linked to survival in these subgroups (Fig. 1D and Table 1).

**Table 1:** Log‐rank *P* values of purine metabolism‐related genes across all patients and in three patient subgroups.

Genes	*P* values for all patients	*P* values in subgroup analysis		
		Wild‐type *TP53*	Mutant *TP53*	MSS
*ADSL*	3.15 × 10^−5^	0.029	0.003	0.04
*APRT*	0.0038	0.04	0.028	0.011
*ADCY3*	3.15 × 10^−6^	0.002	0.014	0.046
*NME3*	0.00079	0.005	0.012	0.004
*NME6*	1.27 × 10^−5^	0.007	0.006	0.011
*PRPS2*	6.16 × 10^−7^	0.023	0.127	0.184
*PPAT*	0.018	0.034	0.767	0.437
*GART*	0.071	0.195	0.879	0.585
*PFAS*	0.002	3.11 × 10^−4^	0.905	0.873
*PAICS*	1.87 × 10^−6^	2.62 × 10^−5^	0.138	0.610
*ATIC*	0.00014	0.069	0.169	0.349
*IMPDH1*	0.0013	0.049	0.222	0.948
*GMPS*	0.00077	0.052	0.073	0.739
*HPRT1*	0.080	0.266	0.271	0.793

Finally, to validate the five common genes (*ADSL*, *APRT*, *ADCY3*, *NME3*, and *NME6*) in independent cohorts, we utilized our own GMC cohort and another publicly available SMC cohort. In the GMC cohort, we performed IHC staining to confirm the clinical relevance by analyzing the association between clinical characteristics and the expression of these five purine metabolic proteins. Additionally, survival analysis was conducted to assess whether these proteins functioned as prognostic biomarkers. In the SMC cohort, we analyzed scRNA‐seq data to identify gene expression changes associated with tumor progression at a single‐cell resolution [38].

### 3.2 Lower gene expression of purine metabolism‐related genes and poor OS across patient subgroups in the GEO cohort

**Fig. 2:**
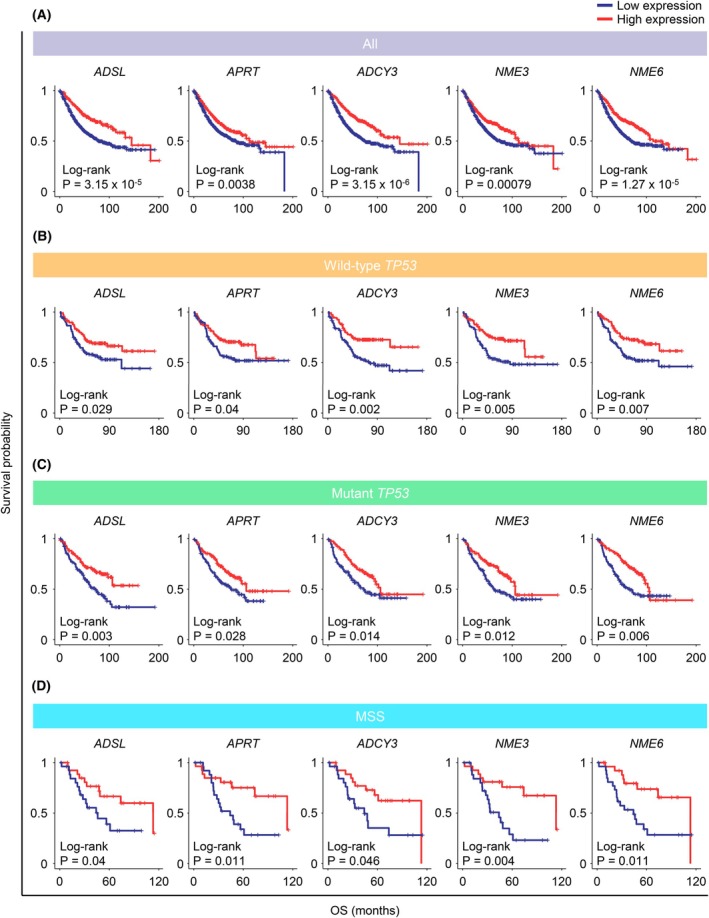
Five purine metabolism‐related genes (i.e., *ADSL*, *APRT*, *ADCY3*, *NME3*, and *NME6*) were significantly associated with prognosis through subgroup analysis of the GEO cohort. Kaplan–Meier curves for OS according to the expression of five purine metabolism‐related genes across all patients with OS (A), wild‐type *TP53* subgroup (B), mutant *TP53* subgroup (C), and MSS subgroup (D) were depicted. GEO, Gene Expression Omnibus; OS, overall survival.

Figure 1C highlights five common purine metabolism‐related genes—*ADSL*, *APRT*, *ADCY3*, *NME3*, and *NME6*—which exhibited statistically significant differences in survival rates (log‐rank *P* < 0.05) between high and low expression groups in the GEO cohort. Kaplan–Meier curves illustrating the correlation between gene expression and OS across all patients are shown in Fig. 2A. Additionally, Kaplan–Meier curves were generated for three CRC patient subgroups—wild‐type *TP53*, mutant *TP53*, and MSS—based on the expression of these five genes. The survival curves for the wild‐type *TP53* group are presented in Fig. 2B, the mutant *TP53* group in Fig. 2C, and the MSS group in Fig. 2D. Across all four CRC patient groups, low expression of the five purine metabolism‐related genes was associated with poor prognosis.

### 3.3 IHC analysis in the GMC cohort

**Fig. 3:**
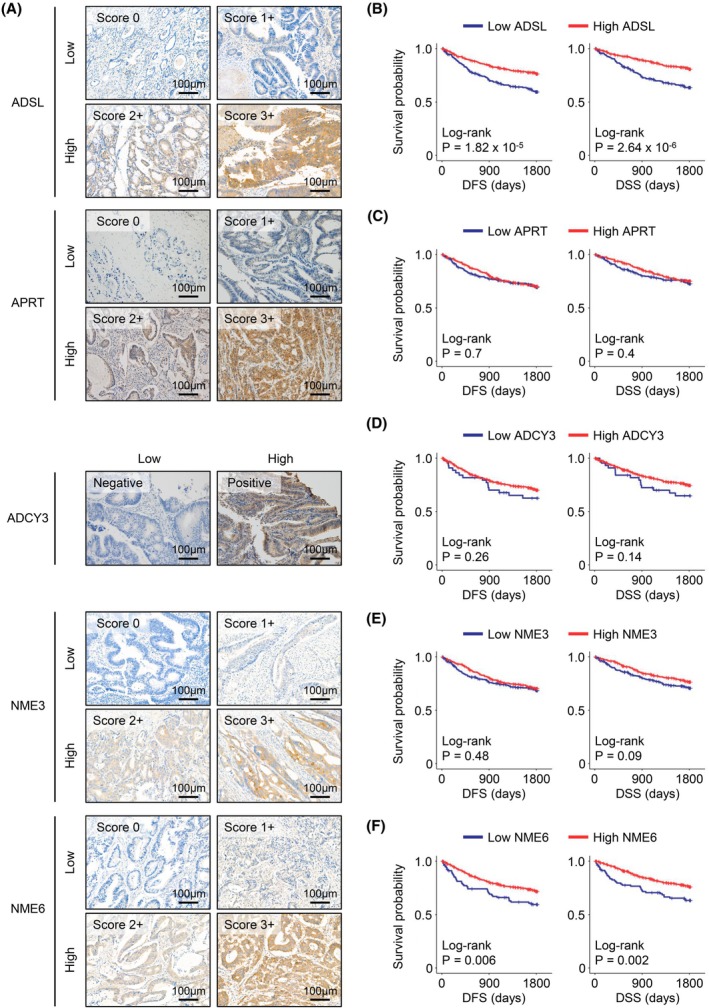
Validation of five purine metabolic proteins as prognostic markers through IHC analysis in the GMC cohort. (A) Representative images of IHC staining for ADSL (*n* = 578), APRT (*n* = 578), ADCY3 (*n* = 581), NME3 (*n* = 582), and NME6 (*n* = 581) on CRC TMA sections. Score 0: no staining. Score 1+: faint, barely discernible cytoplasmic staining in any tumor cells. Score 2+: moderate smooth granular cytoplasmic staining. Score 3+: strong and diffuse cytoplasmic staining. Negative: no staining. Positive: focal or diffuse granular cytoplasmic staining. Original magnification, 200×. Scale bar = 100 μm. (B–F) Kaplan–Meier curves for 5‐year DFS and DSS according to the expression of ADSL (B), APRT (C), ADCY3 (D), NME3 (E), and NME6 (F) in the GMC cohort. CRC, colorectal cancer; DFS, disease‐free survival; DSS, disease‐specific survival; GMC, Gil Medical Center; IHC, immunohistochemistry; TMA, tissue microarray.

To validate the association between survival and the five‐purine metabolism‐related genes, and to explore the clinical significance of purine metabolism‐related protein expression and clinical variables, we established a GMC cohort. We assessed protein expression using IHC staining in 590 patients with CRC within this cohort (Fig. 3A).

### 3.4 Association of purine metabolic proteins with clinical variables in the GMC cohort

In our GMC cohort, the expression of APRT and NME3 was found to be independent of clinical variables, whereas ADSL, ADCY3, and NME6 showed associations with clinical variables. To analyze these associations, we first reviewed the clinical characteristics of the 590 CRC patients, as summarized in Table 2. We employed a univariate analysis using the chi‐squared test to explore the relationships between the expression levels of the five purine metabolic proteins and various clinical variables, including age, sex, smoking history, family history, diabetes, anemia, differentiation, T stage, lymph node metastasis, and distant metastasis. Following this, we selected the clinical variables significantly associated with protein expression in the univariate analysis for further investigation through multivariate logistic regression analysis. This analysis aimed to determine whether the expression of each protein remained clinically significant when adjusted for the selected clinical variables (Table 3).

**Table 2:** Baseline characteristics of the GMC cohort. GMC, Gil Medical Center.

Variables	Subgroups	*N* (%)
Sex	Female	222 (37.63)
	Male	368 (62.37)
Age (years)	< 65	268 (45.42)
	≥ 65	322 (54.58)
Smoking	No	485 (82.20)
	Yes	105 (17.80)
Family history	No	568 (96.27)
	Yes	22 (3.73)
Diabetes	No	485 (82.20)
	Yes	105 (17.80)
Anemia	No	299 (50.68)
	Yes	290 (49.15)
	Unknown	1 (0.17)
Differentiation	Differentiated	548 (92.88)
	Undifferentiated	41 (6.95)
	Unknown	1 (0.17)
T stage	0	6 (1.02)
	1, 2	312 (52.88)
	3, 4	272 (46.10)
Lymph node metastasis	No	329 (55.76)
	Yes	261 (44.24)
Distant metastasis	No	518 (87.80)
	Yes	72 (12.20)

**Table 3:** Associations between clinical variables and the expression of five purine metabolism proteins in univariate and multivariate analyses. *P* values are provided for univariate analysis, and a statistical summary consisting of odds ratio, *P* value, and 95% confidence interval is provided for multivariate analysis. Univariate: univariate analysis (chi‐squared test); multivariate: multivariate logistic regression by taking statistically significant clinical variables (in univariate analysis) and protein expression as explanatory and response variables, respectively; reference; *P < 0.05. ‘–’, excluded in explanatory variables for multivariate analysis due to lack of statistical significance in univariate analysis; NA, not applicable to multivariate analysis due to the number of explanatory variables less than two; NS, nonsignificant in multivariate analysis.

Clinical variables	ADSL (high vs. low)		APRT (high vs. low)		ADCY3 (high vs. low)		NME3 (high vs. low)		NME6 (high vs. low)	
	Univariate	Multivariate	Univariate	Multivariate	Univariate	Multivariate	Univariate	Multivariate	Univariate	Multivariate
Sex										
Female (ref.)	0.605	–	0.967	–	0.625	–	0.778	–	0.245	
Male										
Age										
< 65 years (ref.)	0.686	–	0.379	–	0.521	–	0.198	–	0.135	
≥ 65 years										
Smoking										
No (ref.)	0.715	–	0.317	–	0.355	–	0.413	–	0.298	
Yes										
Family history										
No (ref.)	0.417	–	0.569	–	0.609	–	0.435	–	0.612	
Yes										
Diabetes										
No (ref.)	0.615	–	0.844	–	0.403	–	0.302	–	0.474	
Yes										
Anemia										
No (ref.)	0.745	–	0.132	–	0.538	–	0.504	–	0.061	
Yes										
Differentiation										
No (ref.)	0.101	–	0.070	–	0.071	–	0.182	–	9.69e‐07*	0.25; 0.0001*; 0.13–0.51
Yes										
T stage										
1 & 2 (ref.)	0.083	–	0.799	–	0.245	–	0.074	–	0.157	
3 & 4										
Lymph node metastasis										
No (ref.)	0.003*	0.70; 0.046*; 0.49–0.99	0.578	–	0.327	–	0.951	–	0.015*	NS
Yes										
Distant metastasis										
No (ref.)	0.0004*	0.48; 0.007*; 0.28–0.81	0.066	–	0.008*	NA	0.593	–	0.025*	NS
Yes										

The results indicated that lymph node metastasis and distant metastasis were independent predictors of ADSL expression (lymph node metastasis: OR = 0.70; 95% CI: 0.49–0.99; *P* = 0.046; distant metastasis: OR = 0.48; 95% CI: 0.28–0.81; *P* = 0.007) (Table 3). Differentiation emerged as an independent predictor of NME6 expression (OR = 0.25; 95% CI: 0.13–0.51; *P* = 0.0001) (Table 3). Conversely, APRT, ADCY3, and NME3 did not show independent associations with the clinical variables, suggesting their expression is linked to, but not independently predictive of these variables.

### 3.5 Correlation of purine metabolic proteins with prognosis in the GMC cohort

To further evaluate the clinical relevance of the five purine metabolic proteins, we assessed their correlation with patient prognosis. Survival curves for 5‐year disease‐free survival (DFS) and disease‐specific survival (DSS) were generated based on the expression levels of each protein in the 590 patients (Fig. 3B–F). The survival analysis indicated that patients with low expression of the five purine metabolic proteins generally had poorer survival rates compared to those with high expression. Notably, low expression of ADSL and NME6 was significantly associated with poor prognosis (log‐rank *P* < 0.05) (Fig. 3B,F). ADSL and NME6 emerged as significant poor prognostic factors for both 5‐year DFS and DSS. Although not statistically significant, NME3 may also be a potential prognostic factor for 5‐year DSS, as the Kaplan–Meier curves for DFS and DSS showed similar trends (Fig. 3E).

Since the survival probability of the five purine metabolism‐related genes showed significant difference in MSS and *TP53* subgroup patients (Fig. 2) in the GEO cohort, we inspected, in our IHC results from the GMC cohort, other correlations with MSS or *TP53* status in patients. In the IHC results of the GMC cohort, we performed survival analyses for 5‐year DFS and DSS by classifying the patients into three subgroups: wild‐type *TP53* (*n* = 403), mutant *TP53* (*n* = 113), and MSS (*n* = 264) (Fig. S1). Survival curves for 5‐year DFS were generated based on the expression levels of each protein within the wild‐type *TP53*, mutant *TP53*, and MSS subgroups (Fig. S1A–C). Similarly, survival curves for 5‐year DSS were generated for the same subgroups (Fig. S1D–F).

In all subgroups, low expression of ADSL was significantly associated with poor prognosis (log‐rank *P* < 0.05). Low expression of NME6 was also significantly linked to poor prognosis in the wild‐type *TP53* and MSS subgroups (log‐rank *P* < 0.05). Although some proteins did not achieve statistical significance, a consistent trend was observed across all five proteins, with low‐expression patients exhibiting lower survival rates compared with high‐expression patients.

### 3.6 Subgroup analysis in the GMC cohort: NME3 serves as an early‐stage biomarker whereas ADSL and NME6 in CRC as late‐stage

**Fig. 4:**
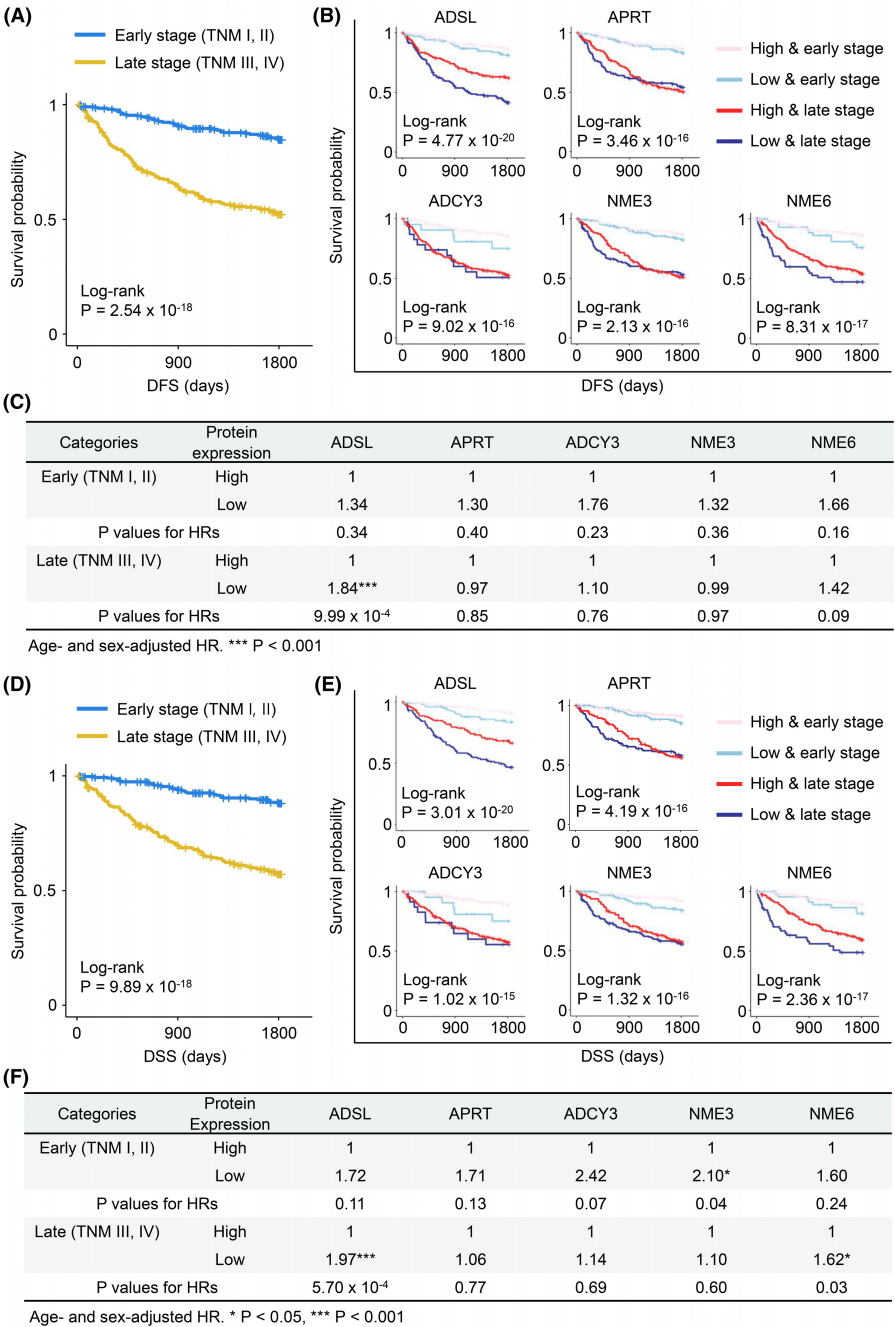
Some purine metabolic proteins can predict high‐risk groups among patients with CRC of early and late TNM stage. (A) Kaplan–Meier curves of early and late‐stage patients for 5‐year DFS. (B) Kaplan–Meier curves for 5‐year DFS of the four patient groups based on TNM stage and protein expression. (C) Age‐ and sex‐adjusted HR of the low‐expressing group of five purine metabolic proteins in early and late‐stage patients for 5‐year DFS. In early‐stage patients, *P* values of the HRs of the low‐expressing groups for ADSL, APRT, ADCY3, NME3, and NME6 were 0.34, 0.40, 0.23, 0.36, and 0.16, respectively. In late‐stage patients, *P* values of the HRs of the low‐expressing groups for ADSL, APRT, ADCY3, NME3, and NME6 were 9.99 × 10^−4^, 0.85, 0.76, 0.97, and 0.09, respectively. The Cox proportional hazards model was used for statistical significance. (D) Kaplan–Meier curves of early and late‐stage patients for 5‐year DSS. (E) Kaplan–Meier curves for 5‐year DSS of the four patient groups based on TNM stage and protein expression. (F) Age‐ and sex‐adjusted HR of the low‐expressing group of five purine metabolic proteins in early and late‐stage patients for 5‐year DSS. In early‐stage patients, *P* values of the HRs of the low‐expression groups for ADSL, APRT, ADCY3, NME3, and NME6 were 0.11, 0.13, 0.07, 0.04, and 0.24, respectively. In late‐stage patients, *P* values of the HRs of the low‐expression groups for ADSL, APRT, ADCY3, NME3, and NME6 were 5.70 × 10^−4^, 0.77, 0.69, 0.60, and 0.03, respectively. The Cox proportional hazards model was utilized to assess statistical significance. CRC, colorectal cancer; DFS, disease‐free survival; DSS, disease‐specific survival; HR, hazard ratio. *P < 0.05, ***P < 0.001.

To evaluate whether the expression levels of five purine metabolic proteins could predict high‐risk groups among patients with early‐ and late‐stage CRC, we analyzed 590 patients with CRC from the GMC cohort. Patients were divided based on the TNM stage: those with TNM stage I or II were classified into the early‐stage group, and those with TNM stage III or IV were classified into the late‐stage group. Kaplan–Meier curves were constructed to assess 5‐year DFS and DSS for both groups (Fig. 4A,D). Additionally, patients were categorized into four groups: low expression in the early stage, high expression in the early stage, low expression in the late stage, and high expression in the late stage. Survival rates for these four groups were analyzed for 5‐year DFS and DSS (Fig. 4B,E).

We used the Cox proportional hazards model to estimate the HRs for differences between low‐ and high‐expression groups within each stage. Age‐ and sex‐adjusted HRs for the five proteins were calculated for each stage group (Tables S4–S13).

For DFS, the adjusted HR for low ADSL expression in the late stage was significantly higher than that of the high expression group in the late stage (HR = 1.84; 95% CI of HR: 1.28–2.65; *P* < 0.001) (Fig. 4C and Table S5).

For DSS, the adjusted HR for low NME3 expression in the early stage was higher than the high expression group (HR = 2.10; 95% CI: 1.02–4.30; *P* = 0.04) (Fig. 4F and Table S10). In the late stage, low expression of ADSL and NME6 was associated with higher HRs than the high expression group (ADSL: HR = 1.97; 95% CI: 1.34–2.90; *P* < 0.001; NME6: HR = 1.62; 95% CI: 1.06–2.48; *P* = 0.027) (Fig. 4F, Tables S5 and S13).

Overall, NME3 serves as a poor prognostic marker for early‐stage CRC, while ADSL and NME6 are poor prognostic markers for late‐stage CRC.

### 3.7 Time‐dependent ROC analysis in the GEO and GMC cohorts

We conducted time‐dependent ROC analysis to evaluate 1‐, 3‐, and 5‐year OS outcomes in all patients, as well as in the subgroups of wild‐type *TP53*, mutant *TP53*, and MSS from the GEO cohort. ROC curves were generated for all patients and the subgroups of wild‐type *TP53*, mutant *TP53*, and MSS (Fig. S2). In the subgroups other than MSS, *APRT* showed higher the area under the ROC curve (AUC) values for the 5‐year OS outcome compared to those for the 1‐year OS outcome, while *ADSL* and *ADCY3* exhibited lower AUC values for the 5‐year OS outcome than those for the 1‐year OS outcome. In the MSS subgroup, *NME6* demonstrated a higher AUC for the 1‐year OS outcome than that for the 5‐year OS outcome. Also, we performed time‐dependent ROC analyses for 1‐, 3‐, and 5‐year DFS outcomes, generating ROC curves for all patients and the subgroups of wild‐type *TP53*, mutant *TP53*, and MSS in the GMC cohort (Fig. S3). Similarly, we conducted time‐dependent ROC analyses for 1‐, 3‐, and 5‐year DSS outcomes, with ROC curves generated for the same groups in the cohort (Fig. S4). In the prediction of DFS and DSS outcomes, most proteins are likely to have higher AUC values for 1‐year outcomes compared to 5‐year outcomes, but ADSL showed a higher AUC for the 5‐year outcome than for the 1‐year outcome. Regarding DFS and DSS, APRT, NME3 and NME6 demonstrated higher predictive performance for 1‐year outcomes compared with 5‐year outcomes in most groups.

### 3.8 Lower gene expression of *ADSL* , *APRT* , *ADCY3* , and *NME3* in epithelial cells is associated with tumor progression in scRNA‐seq of the SMC cohort

**Fig. 5:**
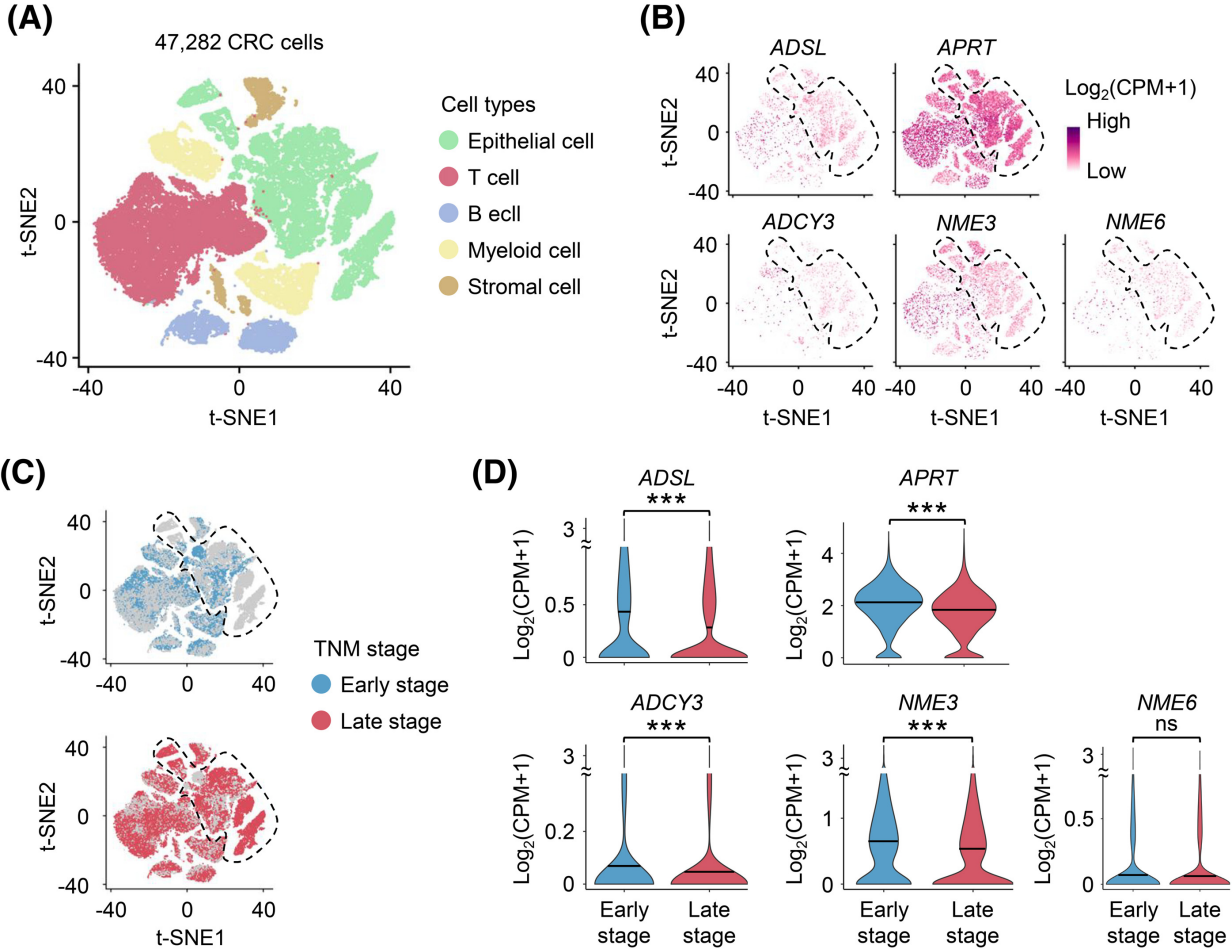
Expression of *ADSL*, *APRT*, *ADCY3*, and *NME3* in epithelial cells was lower in late‐stage CRC than in early‐stage CRC. (A) t‐SNE visualization of 47 282 cells in 23 patients with CRC of the SMC. (B) t‐SNE visualization of the expression of five purine metabolism‐related genes. Epithelial cells are highlighted with dashed lines. (C) t‐SNE visualization of cells of early‐stage CRC patients (blue) and late‐stage CRC patients (red). Epithelial cells are highlighted with dashed lines. (D) Violin plots represent the expression of five purine metabolism‐related genes in epithelial cells of early‐stage and late‐stage CRC patients. Differences between the two groups were evaluated using a Student's *t*‐test. ***P < 0.001. CRC, colorectal cancer; SMC, Samsung Medical Center; t‐SNE, t‐distributed Stochastic Neighbor Embedding.

We analyzed publicly available scRNA‐seq data [38] from CRC to examine the expression of five prognostic purine metabolism‐related genes in CRC progression at a single‐cell resolution. The dataset comprised 47 282 cells derived from the primary tumor tissues of 23 patients with CRC. The cell population included epithelial, T, B, stromal, and myeloid cells (Fig. 5A). Our analysis revealed that the expression of five prognostic purine metabolism‐related genes was notably enriched in epithelial cells (Fig. 5B). To compare gene expression between early‐stage (TNM I and II) and late‐stage (TNM III and IV) epithelial cells, we segregated all cells into early‐ and late‐stage categories (Fig. 5C). The results showed that *ADSL*, *APRT*, *ADCY3*, and *NME3* expression levels were significantly lower (*P* < 0.05) in epithelial cells from late‐stage patients than in early‐stage patients (Fig. 5D). In contrast, the expression of *NME6* did not show a significant difference (Fig. 5D).

### 3.9 Positive correlations between the five survival‐related purine metabolism genes (or proteins) observed in the GEO, GMC, and SMC cohorts

**Fig. 6:**
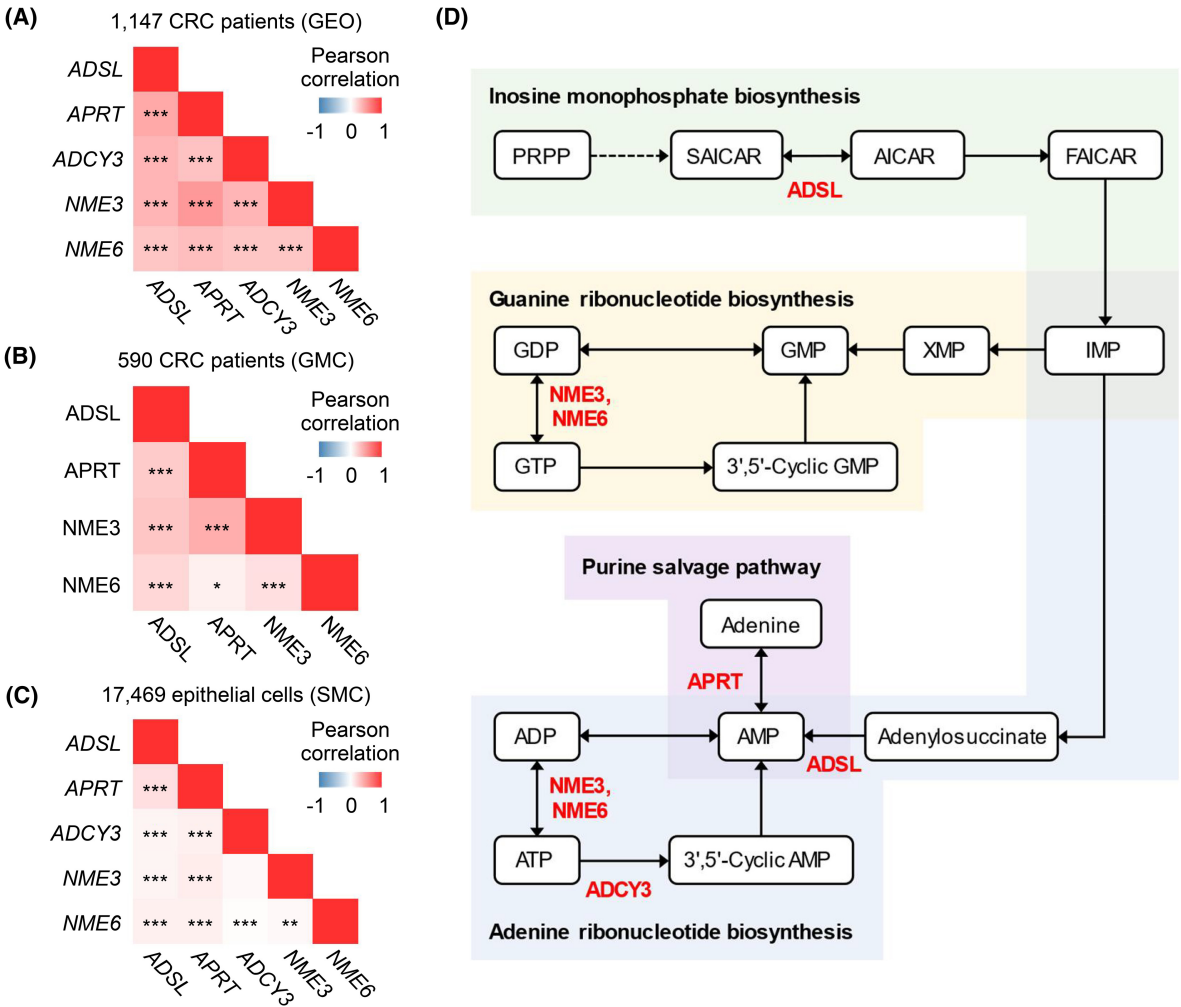
*ADSL*, *APRT*, *ADCY3*, *NME3*, and *NME6* were involved in adenine ribonucleotide biosynthesis in purine metabolism in KEGG. (A) Heatmap of correlation between expression of five purine metabolism‐related genes in 1147 CRC patients from GEO. (B) Heatmap of correlation between expression of four purine metabolic proteins in the GMC cohort. (C) Heatmap of correlation between expression of five purine metabolism‐related genes in 17 469 epithelial cells from the SMC cohort. (D) Schematic diagram of the purine metabolism pathway involving five genes associated with the survival of patients with CRC. The significance on the Pearson's correlation coefficient was tested using a Student's *t*‐distribution. *P < 0.05; **P < 0.01; ***P < 0.001. CRC, colorectal cancer; GEO, Gene Expression Omnibus; GMC, Gil Medical Center; KEGG, Kyoto Encyclopedia of Genes and Genomes; SMC, Samsung Medical Center.

Pearson's correlation analysis was conducted to evaluate the relationships between the gene expression of the five survival‐related purine metabolism genes (*ADSL*, *APRT*, *ADCY3*, *NME3*, and *NME6*) and protein expression among the four purine metabolic proteins. A significant positive correlation was observed between gene expression of the purine metabolism‐related genes in the GEO cohort (*P* < 0.05) (Fig. 6A). In the GMC cohort, a significant positive correlation was found for protein expression of the four purine metabolic proteins (Fig. 6B). In the SMC cohort, which included 17 469 epithelial cells, significant positive correlations were confirmed for all genes except *ADCY3* and *NME3* expression levels (Fig. 6C). Overall, these results affirm that the five prognostic markers are positively correlated.

### 3.10 Five survival‐related purine metabolism genes were involved in adenine ribonucleotide biosynthesis in purine metabolism in Kyoto encyclopedia of genes and genomes (KEGG)

We referred to the KEGG purine metabolism pathway to investigate the functions of the five survival‐related purine metabolism genes (*ADSL*, *APRT*, *ADCY3*, *NME3*, and *NME6*) (Fig. 6D). Our schematic diagram indicated that ADSL is involved in inosine monophosphate biosynthesis (green); NME3 and NME6 are involved in guanine ribonucleotide biosynthesis (yellow); APRT is involved in the purine salvage pathway (purple); and ADSL, NME3, NME6, and ADCY3 participate in adenine ribonucleotide biosynthesis (blue). We identified that these five survival‐related purine metabolism genes are primarily involved in adenine ribonucleotide biosynthesis. Additionally, in CRC tissues, our analysis showed consistent gene expression directionality for these five genes (*ADSL*, *APRT*, *ADCY3*, *NME3*, and *NME6*), despite the reversible nature of reactions in the purine metabolism pathway. These findings (Fig. 6) highlight the consistent regulatory patterns among these genes, which collectively impact adenine ribonucleotide biosynthesis in the context of CRC prognosis.

## 4 Discussion

We identified that the expression of the five purine metabolism‐related genes (*ADSL*, *APRT*, *ADCY3*, *NME3*, and *NME6*) correlated with prognosis in public datasets of patients with CRC. Our subgroup analyses incorporated *TP53* mutation and MSI status because both are well‐established determinants of CRC prognosis and reflect the tumor's biological heterogeneity. *TP53* mutations are linked to genomic instability and treatment resistance, while MSI status indicates distinct tumor immune microenvironments [46]. These criteria allowed us to evaluate purine metabolism's prognostic role in biologically diverse patient subgroups, strengthening the translational relevance of our findings. Low expression levels of these genes were linked to poor prognosis. This association was further validated by IHC, a crucial method for clinical validation that examines both the clinical significance and oncological outcomes (e.g., survival) of biomarkers using surgical specimens. ADSL and NME6 were poor prognostic markers in late‐stage, whereas NME3 was a poor prognostic marker in early‐stage. ADSL, ADCY3, and NME6 were associated with lymph node metastasis, distant metastasis, and differentiation, whereas APRT and NME3 were independent of these clinical variables. Additionally, scRNA‐seq analysis confirmed that the expression levels of *ADSL*, *APRT*, *ADCY3*, and *NME3* in epithelial cells were significantly lower in late‐stage patients than in early‐stage patients.

Our findings indicate that low expression of ADSL and NME6 identifies high‐risk late‐stage TNM CRC patients. ADSL knockout leads to phosphoribosyl aminoimidazole succinocarboxamide accumulation, which binds to pyruvate kinase M2, activating proteins involved in cell proliferation [47, 48]. NME6 regulates oxidative phosphorylation, and its overexpression is linked to increased dysfunction in this process [49]. Colon cancer relies on oxidative phosphorylation and has more mitochondria; inhibiting mitochondrial electron transfer reduces cancer growth [50, 51]. These findings correlate low ADSL and NME6 expression with poorer prognosis in CRC. Purine metabolism's roles in tumor growth and metastasis could provide deeper mechanistic insights. For instance, these genes' dysregulation may disrupt energy balance and nucleotide biosynthesis pathways essential for tumor proliferation.

NME3 protein expression is a promising biomarker for CRC. Low NME3 expression, even in early TNM stages, identifies high‐risk patients. NME3, a subunit of nucleoside diphosphate kinase, is crucial for DNA repair by supplying dNTPs [52, 53]. Dysregulated NME3 expression in CRC is linked to increased invasion and metastasis [54].

Given that invasion and metastasis are typically linked to advanced stages [55], low NME3 expression in early‐stage CRC may indicate a more advanced disease state, despite being clinically classified as early stage. The limitations of the current clinical staging system in fully capturing the progression of early‐stage CRC underscore the value of NME3 protein expression. As a complementary tool, NME3 expression could refine early‐stage CRC diagnosis.

ADCY3 catalyzes cyclic AMP (cAMP) synthesis, and its overexpression suppresses CRC cell proliferation [56, 57]. APRT increases AMP levels, activating AMPK, which inhibits cancer growth [58, 59]. Reportedly, AMPK knockdown in CRC cells restores cell survival and reduces apoptosis [60]. Downregulation of ADCY3 and APRT decreases cAMP and AMPK signaling, potentially increasing CRC cell proliferation [56, 58, 59], linking low expression of these genes to poor CRC prognosis.

While the link between purine metabolic proteins and prognosis via IHC is not well‐established [16, 61], our study found that low expression of ADSL, NME3, and NME6 correlates with poor CRC prognosis. Notably, NME3 emerged as an independent predictor of poor outcomes, capable of forecasting prognosis regardless of clinical variables that determine tumor aggressiveness. In particular, among early‐stage patients, low NME3 expression indicates a higher risk of recurrence and poor survival outcomes (Fig. 4F and Table S10). Thus, NME3 helps identify those who may benefit from more aggressive treatments, such as adjuvant therapies. The translational relevance of these findings highlights their potential to guide personalized treatment strategies, especially in identifying early‐stage CRC patients who may benefit from adjuvant therapies.

We found that *ADSL*, *APRT*, *ADCY3*, and *NME3* are expressed in epithelial cells, showing lower expression in late‐stage CRC patients compared to early‐stage patients. While no previous studies have reported this association in epithelial cells via scRNA‐seq, prior research identified such associations in tumor‐associated macrophages from CRC patients [62]. Our results are significant as they show consistent findings between scRNA‐seq and IHC for several purine metabolism‐related markers.

Our study did not delve deeply into cellular mechanisms, highlighting the need for further *in vitro* and *in vivo* experiments. Despite these limitations, we identified several novel purine metabolism‐related prognostic biomarkers in CRC. A key strength of our study is the use of translational research methods, emphasizing the clinical relevance of these biomarkers. We validated them using cost‐effective and easily applicable techniques, particularly IHC, which is readily implementable in clinical settings without specialized equipment [19, 20]. This underscores the potential for our findings to be swiftly integrated into clinical practice.

## 5 Conclusions

We identified ADSL, APRT, ADCY3, NME3, and NME6 as clinically significant prognostic markers in CRC. IHC validation revealed that ADSL and NME6 are poor prognostic markers associated with late‐stage disease and key clinical variables, including differentiation and metastasis. Conversely, NME3 emerged as an independent prognostic marker specifically for early‐stage CRC, highlighting its potential to identify high‐risk patients requiring more aggressive treatment. These findings suggest that purine metabolism‐related proteins hold promise as both prognostic biomarkers and targets for understanding tumor‐suppressive mechanisms in CRC.

## Conflict of interest

The authors declare no conflict of interest.

## Author contributions

Conceptualization, SN and JHK; data curation, SK; formal analysis, SK; funding acquisition, JHK and SN; investigation, SK; methodology, SK, MK, SJ, and JK; data analysis, SK; supervision, JHK, and SN; validation, SK and MK; visualization, SK and MK; roles/writing–original draft, SK, MK, and SN; writing–review & editing, JK, SJ, KOK, W‐SL, J‐HB, SK, MK, JHK, and SN.

## Peer review

The peer review history for this article is available at https://www.webofscience.com/api/gateway/wos/peer‐review/10.1002/1878‐0261.70010.

## Supporting information


**Fig. S1.** Survival analysis according to the expression of five purine metabolic proteins in subgroups of GMC cohort.
**Fig. S2.** Time‐dependent ROC curves of five purine metabolism‐related genes for predicting 1‐, 3‐, 5‐year OS outcome in GEO cohort.
**Fig. S3.** Time‐dependent ROC curves of five purine metabolic proteins for predicting 1‐, 3‐, and 5‐year DFS outcome in GMC cohort.
**Fig. S4.** Time‐dependent ROC curves of five purine metabolic proteins for predicting 1‐, 3‐, and 5‐year DSS outcome in GMC cohort.
**Table S1.** Summary of the GEO cohort.
**Table S2.** Cutoff values of five purine metabolism‐related genes to classify into the low and high expressing group in all patients of GEO cohort.
**Table S3.** Cutoff values of five purine metabolism‐related genes to classify into the low and high expressing group in subgroups of GEO cohort.
**Table S4.** The hazard ratio of low ADSL expression and early TNM stage group adjusted for age and sex in 5‐year DFS and DSS.
**Table S5.** The hazard ratio of low ADSL expression and late TNM stage group adjusted for age and sex in 5‐year DFS and DSS.
**Table S6.** The hazard ratio of low APRT expression and early TNM stage group adjusted for age and sex in 5‐year DFS and DSS.
**Table S7.** The hazard ratio of low APRT expression and late TNM stage group adjusted for age and sex in 5‐year DFS and DSS.
**Table S8.** The hazard ratio of low ADCY3 expression and early TNM stage group adjusted for age and sex in 5‐year DFS and DSS.
**Table S9.** The hazard ratio of low ADCY3 expression and late TNM stage group adjusted for age and sex in 5‐year DFS and DSS.
**Table S10.** The hazard ratio of low NME3 expression and early TNM stage group adjusted for age and sex in 5‐year DFS and DSS.
**Table S11.** The hazard ratio of low NME3 expression and late TNM stage group adjusted for age and sex in 5‐year DFS and DSS.
**Table S12.** The hazard ratio of low NME6 expression and early TNM stage group adjusted for age and sex in 5‐year DFS and DSS.
**Table S13.** The hazard ratio of low NME6 expression and late TNM stage group adjusted for age and sex in 5‐year DFS and DSS.

## Data Availability

The data used in this study are publicly available in GEO (https://www.ncbi.nlm.nih.gov/geo/) database at: accession numbers: GSE161158, GSE72969, GSE72968, GSE38832, GSE39084, GSE29621, GSE39582, GSE30378, GSE31595, GSE24550, GSE24549, GSE17537, GSE17536, GSE16125, GSE12945, GSE106535, and GSE132465.
