# Supplementary material for: Elucidating prognostic significance of purine metabolism in colorectal cancer through integrating data from transcriptomic, immunohistochemical, and single‐cell RNA sequencing analysis
Source: Mol Oncol. 2025 Feb 27;19(8):2310–29. doi: 10.1002/1878-0261.70010 (PMC12330927; doi:10.1002/1878-0261.70010)

Supplementary Information

Elucidating prognostic significance of purine metabolism in colorectal cancer through

integrating data from transcriptomic, immunohistochemical, and single-cell RNA

sequencing analysis

Kim and Kang et al.

Tables S1 through S13

Figures S1 through S4

Table S1. Summary of the GEO cohort. The nine out of 16 datasets were included in our subsequent survival analysis.

Accession

GSE161158

GSE72969

GSE72968

GSE38832

GSE39084

GSE29621

GSE39582

GSE30378

GSE31595

GSE24550

GSE24549

GSE17537

GSE17536

GSE16125

GSE12945

GSE106535

Platform

Samples Country

Age

Sex

NA

TNM stage

Patients with OS

<65 (120),

I (33), II (76),

III (82), IV (59)

Affymetrix Human Genome U133 Plus 2.0 Array

Affymetrix Human Genome U133 Plus 2.0 Array

Affymetrix Human Genome U133 Plus 2.0 Array

Affymetrix Human Genome U133 Plus 2.0 Array

Affymetrix Human Genome U133 Plus 2.0 Array

Affymetrix Human Genome U133 Plus 2.0 Array

Affymetrix Human Genome U133 Plus 2.0 Array

Affymetrix Human Exon 1.0 ST Array

250

56

USA

France

France

USA

NA

56

≥65 (130)

<65 (29),

M (32),

F (24)

M (42),

F (26)

NA

NA

NA

≥65 (27)

<65 (43),

68

68

≥65 (25)

NA

122

70

NA

NA

70

<65 (32),

M (35),

F (35)

M (40),

F (25)

M (310),

F (256)

I (8), II (23),

III (16), IV (22)

France

USA

≥65 (38)

65

NA

NA

65

I (33), II (264),

III (205), IV (60)

I (0), II (6),

III (6), IV (0)

566

12

France

Norway

Denmark

Norway

Norway

USA

NA

562

NA

NA

NA

NA

55

NA

NA

<65 (7),

M (15),

F (22)

Affymetrix Human Genome U133 Plus 2.0 Array

37

NA

≥65 (30)

Affymetrix Human Exon 1.0 ST Array, Human

Exon 1.0 ST Array

I (0), II (88),

III (66), IV (0)

I (0), II (92),

III (74), IV (0)

154

166

55

NA

NA

NA

Affymetrix Human Exon 1.0 ST Array, Human

NA

Exon 1.0 ST Array

<65 (32),

M (26),

F (29)

M (96),

F (81)

M (16),

F (20)

M (34),

F (28)

Affymetrix Human Genome U133 Plus 2.0 Array

Affymetrix Human Genome U133 Plus 2.0 Array

Affymetrix Human Exon 1.0 ST Array

NA

NA

NA

NA

NA

≥65 (23)

<65 (78),

177

36

USA

177

32

≥65 (99)

<65 (15),

Italy

≥65 (21)

<65 (30),

Affymetrix Human Genome U133AArray

Affymetrix Human Exon 1.0 ST Array

62

Germany

62

≥65 (32)

New

Zealand

35

NA

NA

NA

Abbreviations: OS: overall survival; NA: not available; M: male; F: female.


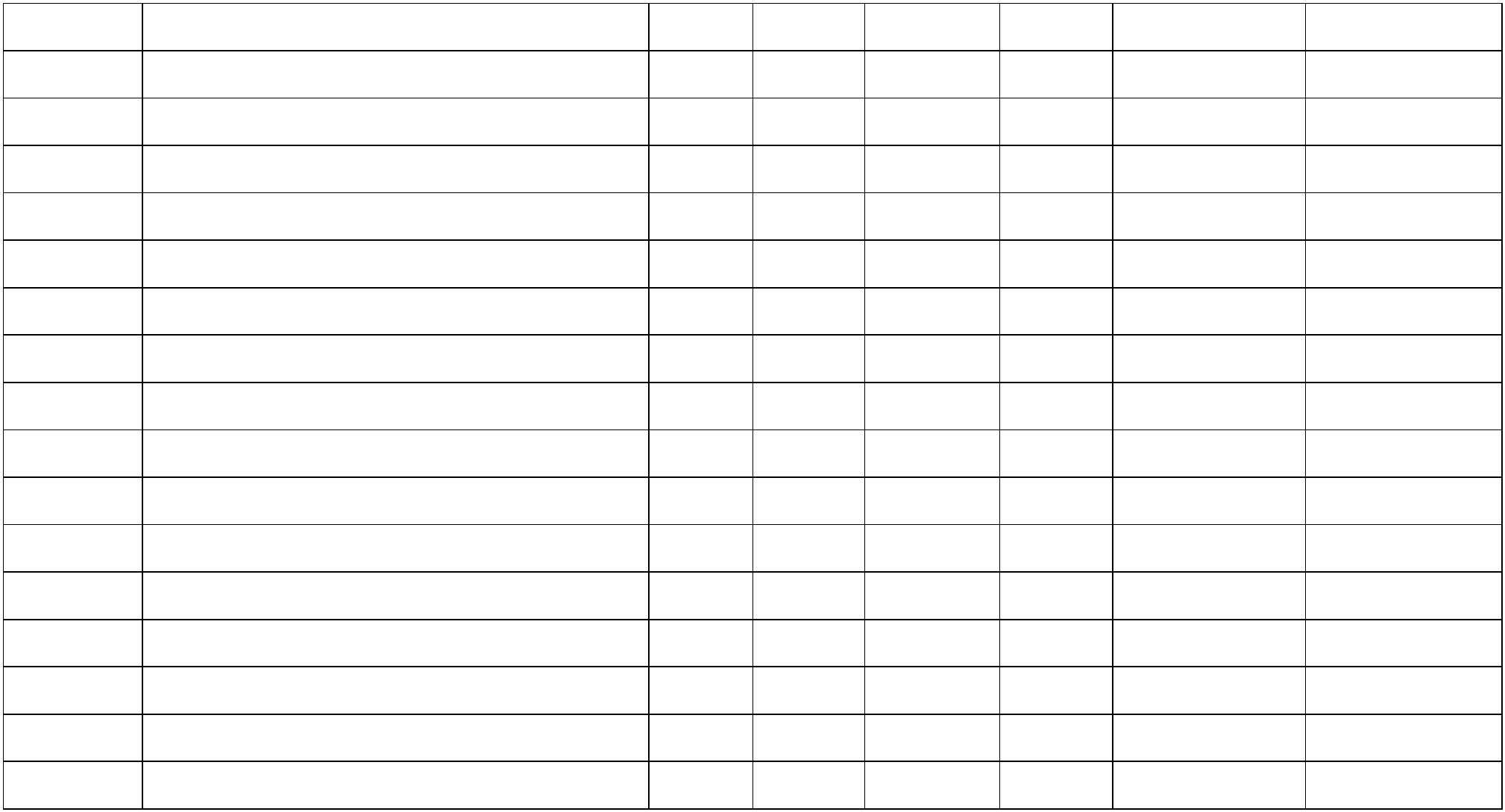


Table S2. Cutoff values of five purine metabolism-related genes to classify into the low and high expressing group

in all patients of GEO cohort.

Genes

ADSL

Method

Cutoff expression levels

10.16024

Optimal cutoff calculated by "surv_miner" package

Optimal cutoff calculated by "surv_miner" package

Optimal cutoff calculated by "surv_miner" package

Optimal cutoff calculated by "surv_miner" package

Optimal cutoff calculated by "surv_miner" package

APRT

9.56381

ADCY3

NME3

NME6

7.839211

8.368325

7.570355


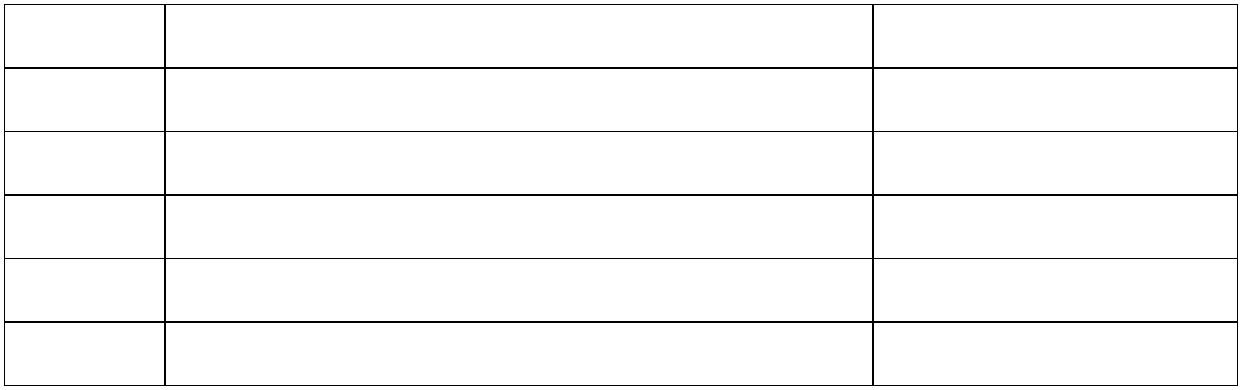


Table S3. Cutoff values of five purine metabolism-related genes to classify into the low and high expressing group

in subgroups of GEO cohort.

Genes

Subgroups

Method

Cutoff expression levels

Wild-type TP53

Mutant TP53

MSS

Median

Median

Median

Median

Median

Median

Median

Median

Median

Median

Median

Median

Median

Median

Median

9.953949

9.941887

9.837523

9.426781

9.487296

9.338256

7.62144

ADSL

Wild-type TP53

Mutant TP53

MSS

APRT

ADCY3

NME3

NME6

Wild-type TP53

Mutant TP53

MSS

7.768751

7.688439

8.393984

8.306865

8.26296

Wild-type TP53

Mutant TP53

MSS

Wild-type TP53

Mutant TP53

MSS

7.59078

7.567454

7.368059


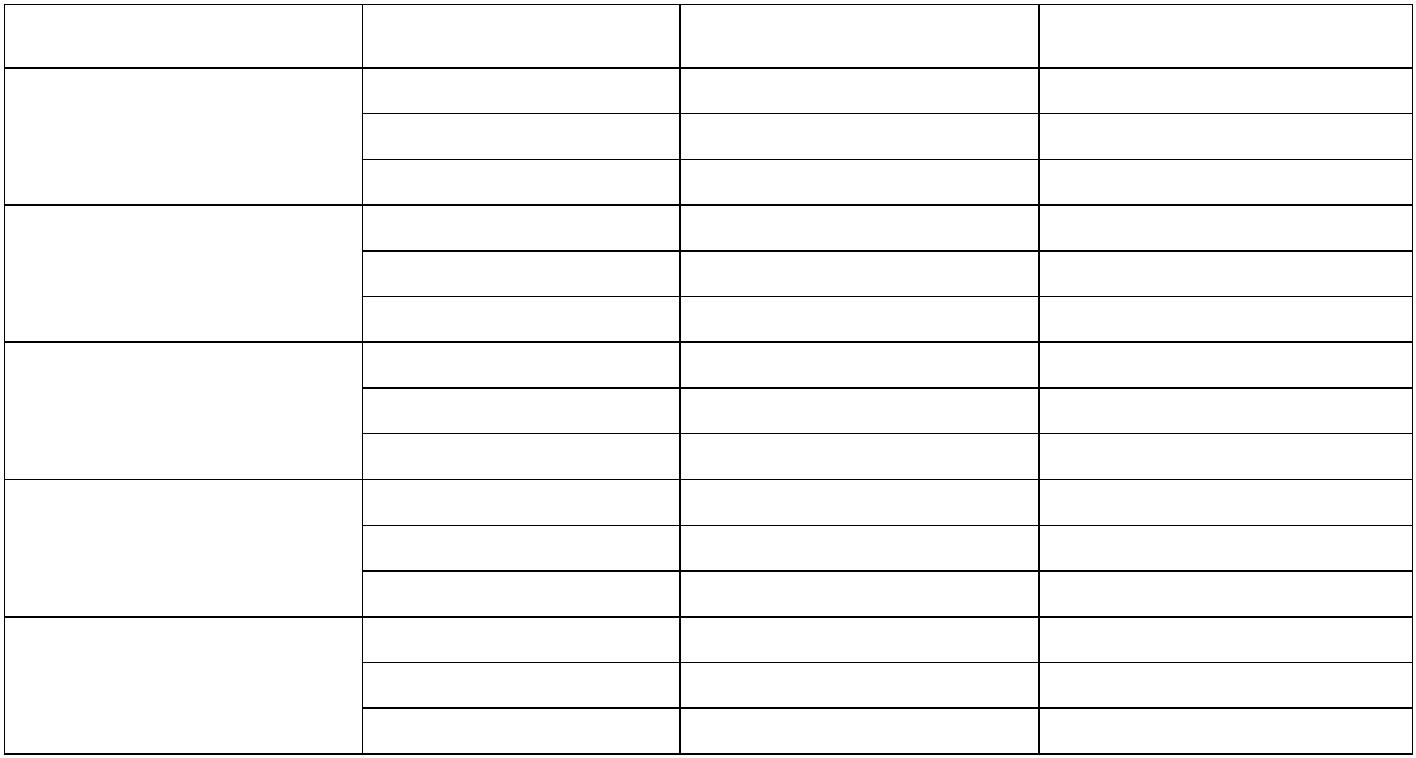


Table S4. The hazard ratio of low ADSL expression & early TNM stage group adjusted for age and sex in 5-year DFS and DSS

DFS

DSS

Variables

HR (95% CI)

P value

0.341

HR (95% CI)

1

P value

0.11

Protein expression & early TNM stage

High ADSL expression & early TNM stage

1

Low ADSL expression & early TNM stage

1.34 (0.73–2.44)

1.72 (0.88–3.37)

Age (years)

< 65

1

1

≥ 65

1.97 (1.42–2.72)

< 0.001

0.009

2.03 (1.43–2.89)

< 0.001

0.08

Sex

Female

Male

1

1

1.55 (1.11–2.15)

1.37 (0.97–1.94)

Abbreviations: ADSL, adenylosuccinate lyase; DFS, disease-free survival; DSS, disease-specific survival; HR, hazard ratio; CI, confidence interval.


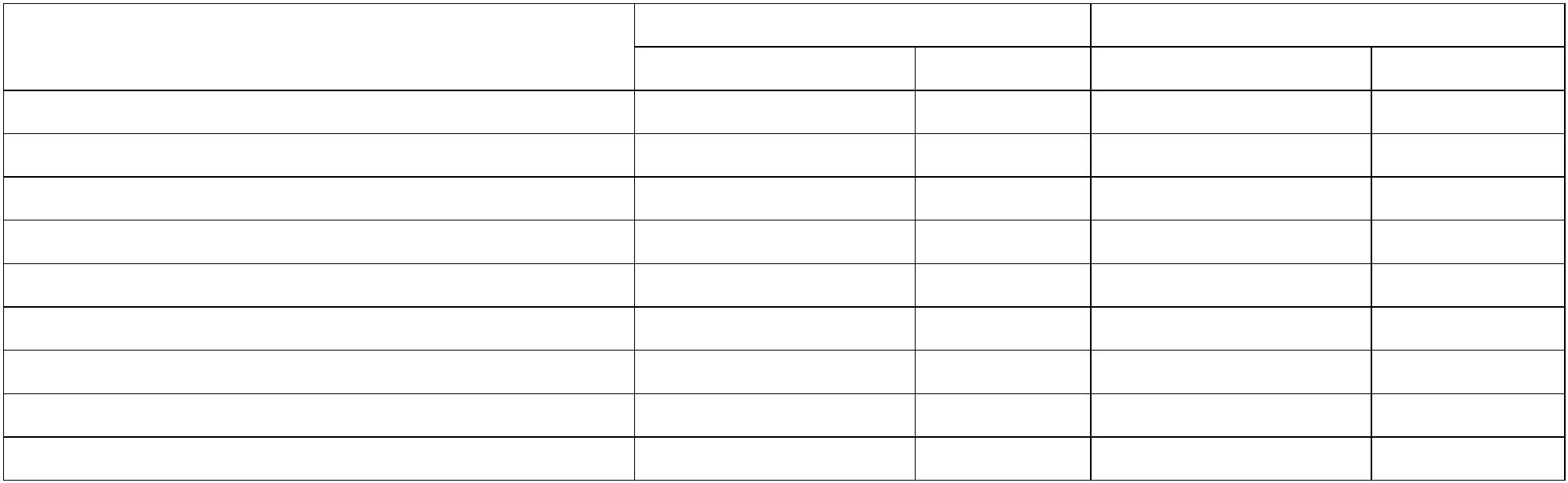


Table S5. The hazard ratio of low ADSL expression & late TNM stage group adjusted for age and sex in 5-year DFS and DSS

DFS

DSS

Variables

HR (95% CI)

P value

< 0.001

< 0.001

0.009

HR (95% CI)

1

P value

< 0.001

< 0.001

0.08

Protein expression & late TNM stage

High ADSL expression & late TNM stage

1

Low ADSL expression & late TNM stage

1.84 (1.28–2.65)

1.97 (1.34–2.90)

Age (years)

< 65

1

1

≥ 65

1.97 (1.42–2.72)

2.03 (1.43–2.89)

Sex

Female

Male

1

1

1.55 (1.11–2.15)

1.37 (0.97–1.94)

Abbreviations: ADSL, adenylosuccinate lyase; DFS, disease-free survival; DSS, disease-specific survival; HR, hazard ratio; CI, confidence interval.


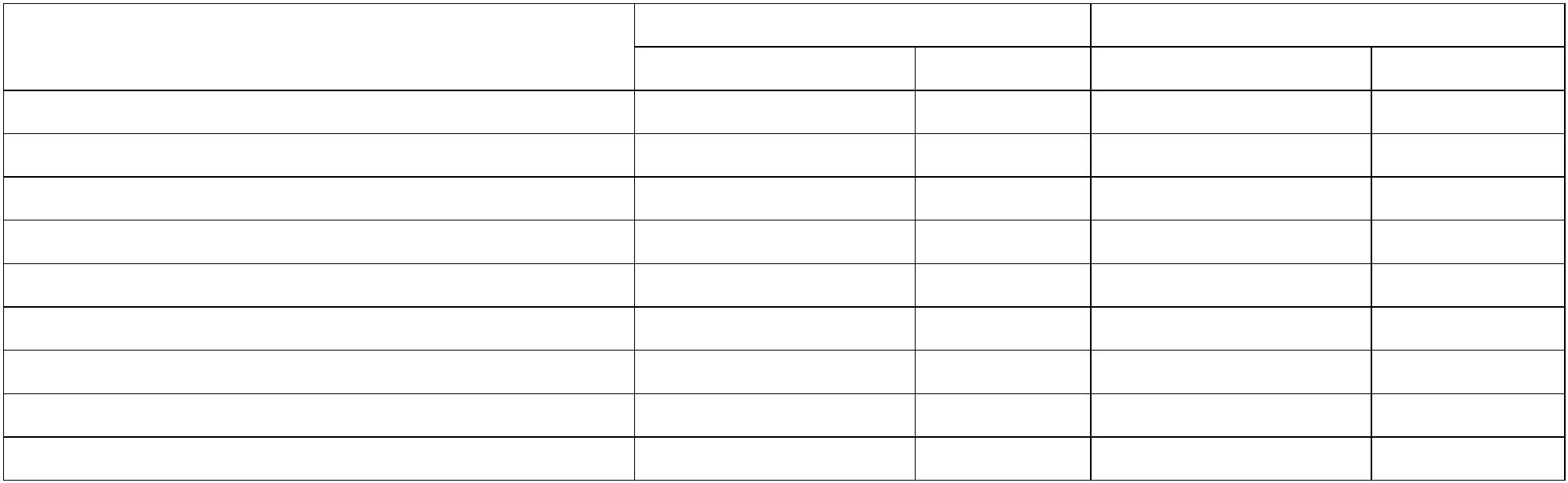


Table S6. The hazard ratio of low APRT expression & early TNM stage group adjusted for age and sex in 5-year DFS and DSS

DFS

DSS

Variables

HR (95% CI)

P value

0.393

HR (95% CI)

1

P value

0.13

Protein expression & early TNM stage

High APRT expression & early TNM stage

1

Low APRT expression & early TNM stage

1.30 (0.71–2.37)

1.71 (0.85–3.43)

Age (years)

< 65

1

1

≥ 65

1.95 (1.41–2.69)

< 0.001

0.008

1.99 (1.40–2.84)

< 0.001

0.08

Sex

Female

Male

1

1

1.56 (1.12–2.17)

1.37 (0.96–1.94)

Abbreviations: APRT, adenine phosphoribosyltransferase; DFS, disease-free survival; DSS, disease-specific survival; HR, hazard ratio; CI, confidence interval.


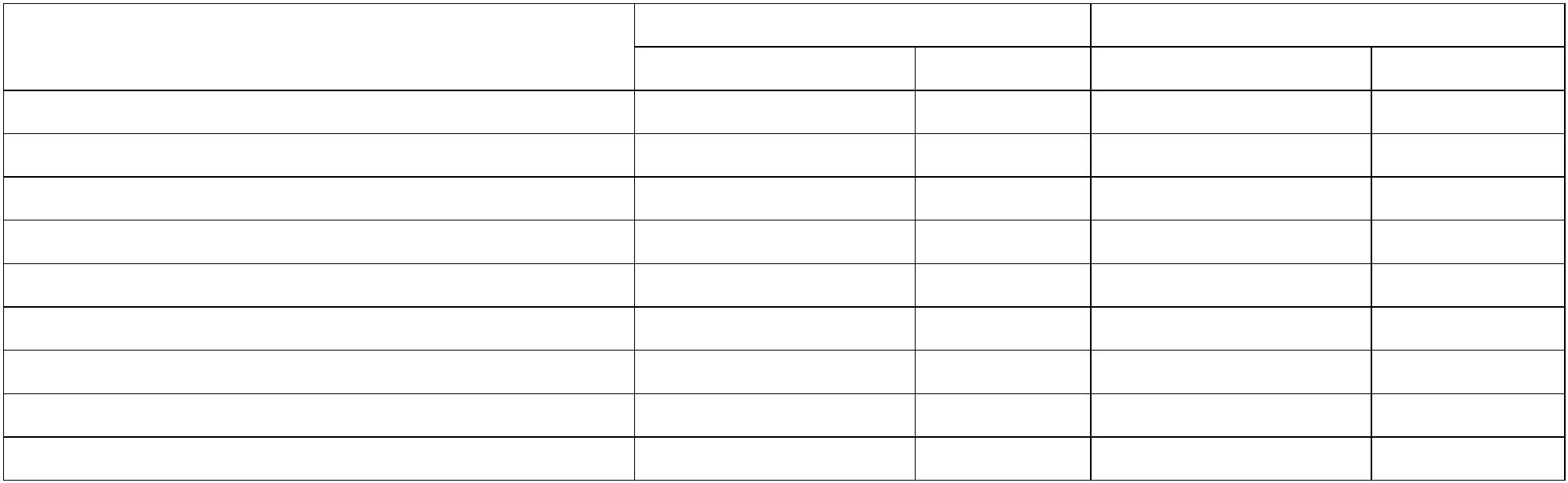


Table S7. The hazard ratio of low APRT expression & late TNM stage group adjusted for age and sex in 5-year DFS and DSS

DFS

DSS

Variables

HR (95% CI)

P value

0.847

HR (95% CI)

1

P value

0.77

Protein expression & late TNM stage

High APRT expression & late TNM stage

1

Low APRT expression & late TNM stage

0.97 (0.68–1.38)

1.06 (0.72–1.54)

Age (years)

< 65

1

1

≥ 65

1.95 (1.41–2.69)

< 0.001

0.008

1.99 (1.40–2.84)

< 0.001

0.08

Sex

Female

Male

1

1

1.56 (1.12–2.17)

1.37 (0.96–1.94)

Abbreviations: APRT, adenine phosphoribosyltransferase; DFS, disease-free survival; DSS, disease-specific survival; HR, hazard ratio; CI, confidence interval.


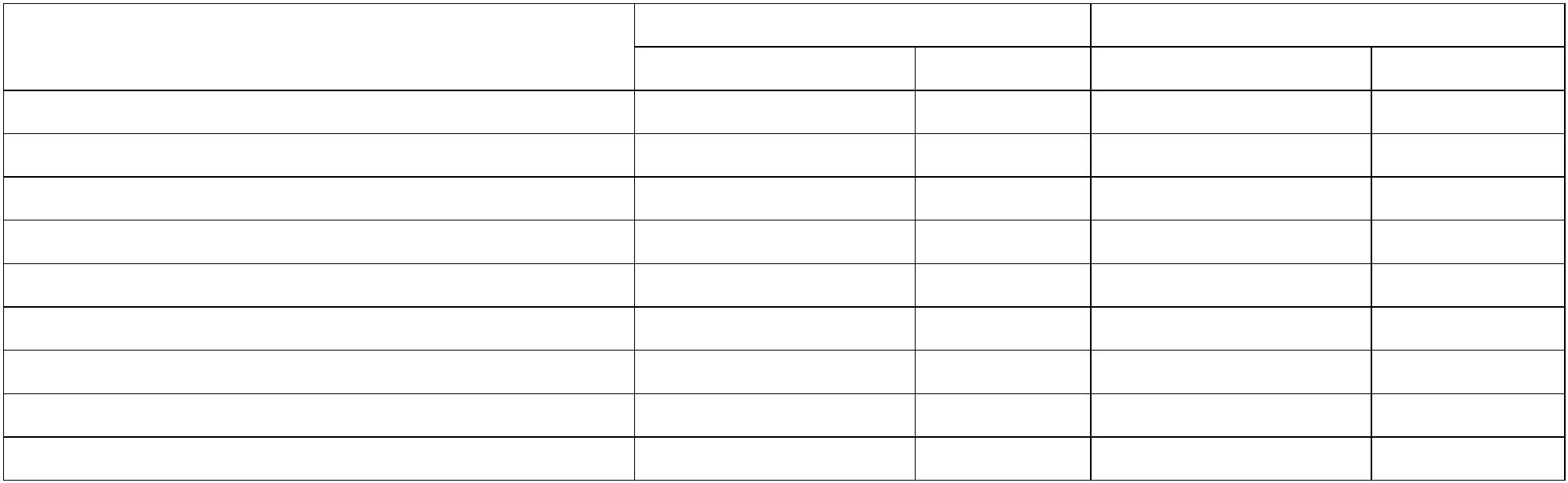


Table S8. The hazard ratio of low ADCY3 expression & early TNM stage group adjusted for age and sex in 5-year DFS and DSS

DFS

DSS

Variables

HR (95% CI)

P value

0.232

HR (95% CI)

1

P value

0.07

Protein expression & early TNM stage

High ADCY3 expression & early TNM stage

1

Low ADCY3 expression & early TNM stage

1.76 (0.70–4.48)

2.42 (0.94–6.26)

Age (years)

< 65

1

1

≥ 65

1.92 (1.39–2.65)

< 0.001

0.007

1.96 (1.39–2.79)

< 0.001

0.07

Sex

Female

Male

1

1

1.57 (1.13–2.18)

1.38 (0.97–1.95)

Abbreviations: ADCY3, adenylate cyclase 3; DFS, disease-free survival; DSS, disease-specific survival; HR, hazard ratio; CI, confidence interval.


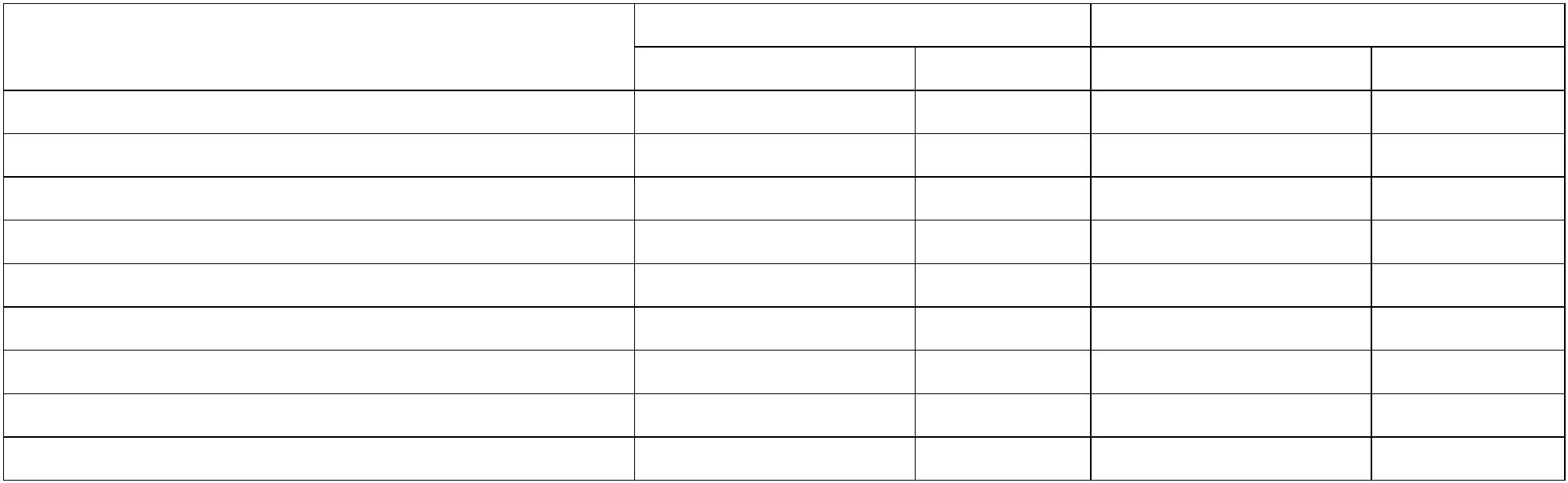


Table S9. The hazard ratio of low ADCY3 expression & late TNM stage group adjusted for age and sex in 5-year DFS and DSS

DFS

DSS

Variables

HR (95% CI)

P value

0.761

HR (95% CI)

1

P value

0.69

Protein expression & late TNM stage

High ADCY3 expression & late TNM stage

1

Low ADCY3 expression & late TNM stage

1.10 (0.59–2.05)

1.14 (0.60–2.19)

Age (years)

< 65

1

1

≥ 65

1.92 (1.39–2.65)

< 0.001

0.007

1.96 (1.39–2.79)

< 0.001

0.07

Sex

Female

Male

1

1

1.57 (1.13–2.18)

1.38 (0.97–1.95)

Abbreviations: ADCY3, adenylosuccinate lyase; DFS, disease-free survival; DSS, disease-specific survival; HR, hazard ratio; CI, confidence interval.


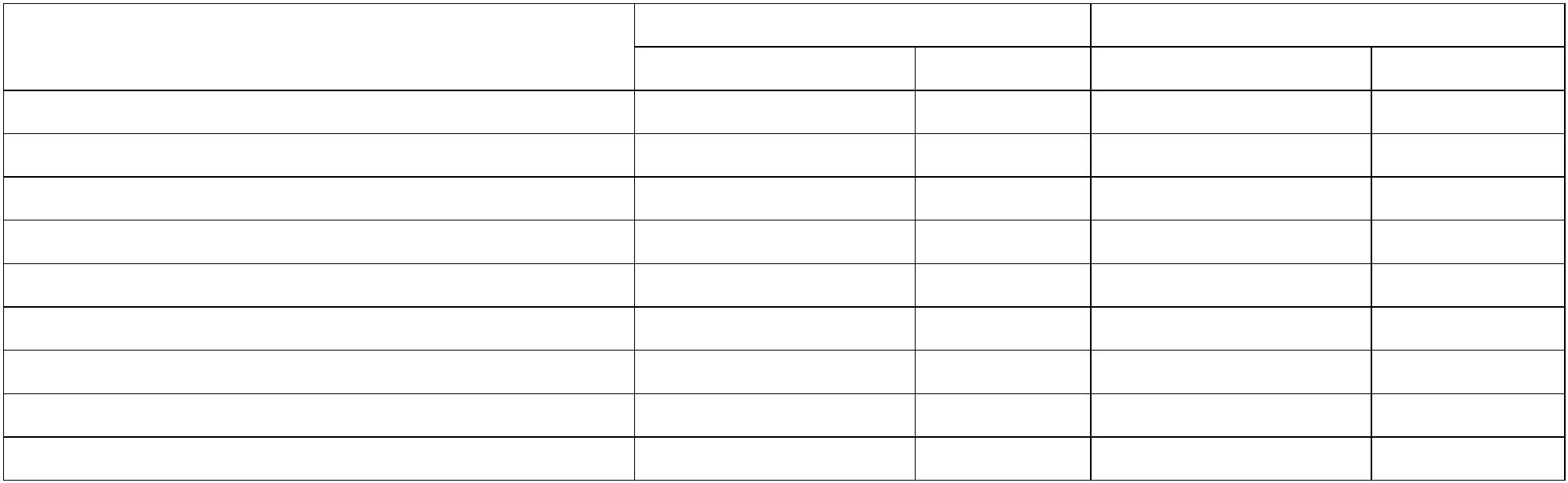


Table S10. The hazard ratio of low NME3 expression & early TNM stage group adjusted for age and sex in 5-year DFS and DSS

DFS

DSS

Variables

HR (95% CI)

P value

0.361

HR (95% CI)

1

P value

0.04

Protein expression & early TNM stage

High NME3 expression & early TNM stage

1

Low NME3 expression & early TNM stage

1.32 (0.73–2.40)

2.10 (1.02–4.30)

Age (years)

< 65

1

1

≥ 65

1.94 (1.41–2.68)

< 0.001

0.009

1.97 (1.39–2.80)

< 0.001

0.09

Sex

Female

Male

1

1

1.55 (1.11–2.14)

1.36 (0.96–1.92)

Abbreviations: NME3, nucleoside diphosphate kinase 3; DFS, disease-free survival; DSS, disease-specific survival; HR, hazard ratio; CI, confidence interval.


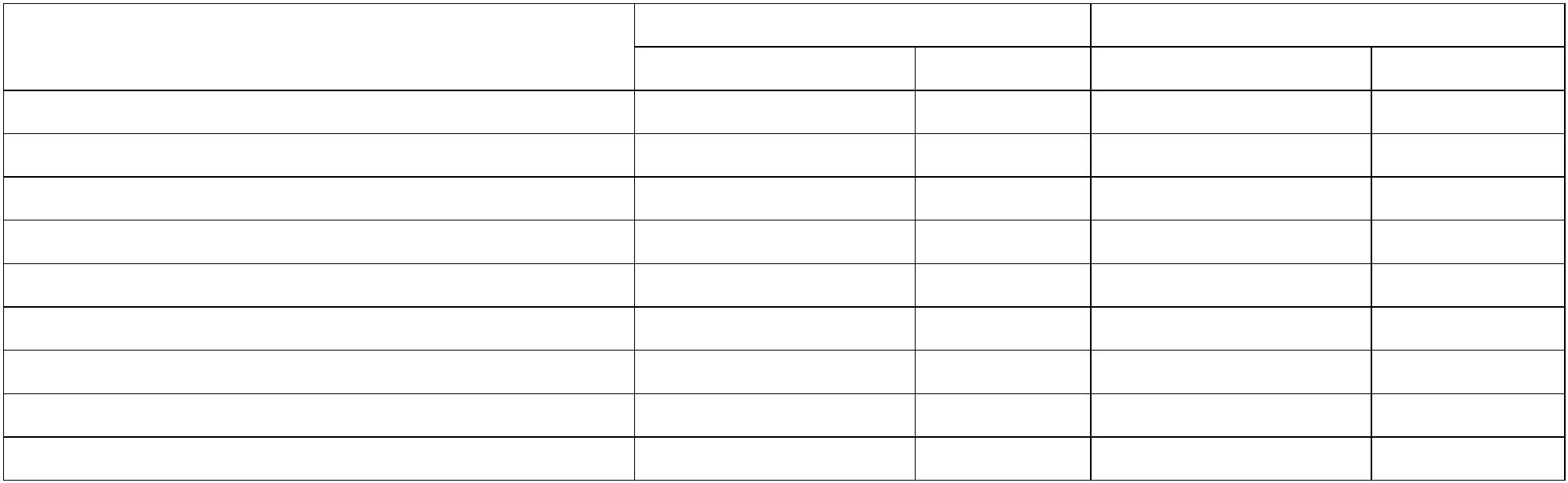


Table S11. The hazard ratio of low NME3 expression & late TNM stage group adjusted for age and sex in 5-year DFS and DSS

DFS

DSS

Variables

HR (95% CI)

P value

0.972

HR (95% CI)

1

P value

0.60

Protein expression & late TNM stage

High NME3 expression & late TNM stage

1

Low NME3 expression & late TNM stage

0.99 (0.70–1.42)

1.10 (0.76–1.61)

Age (years)

< 65

1

1

≥ 65

1.94 (1.41–2.68)

< 0.001

0.009

1.97 (1.39–2.80)

< 0.001

0.09

Sex

Female

Male

1

1

1.55 (1.11–2.14)

1.36 (0.96–1.92)

Abbreviations: NME3, nucleoside diphosphate kinase 3, disease-free survival; DSS, disease-specific survival; HR, hazard ratio; CI, confidence interval.


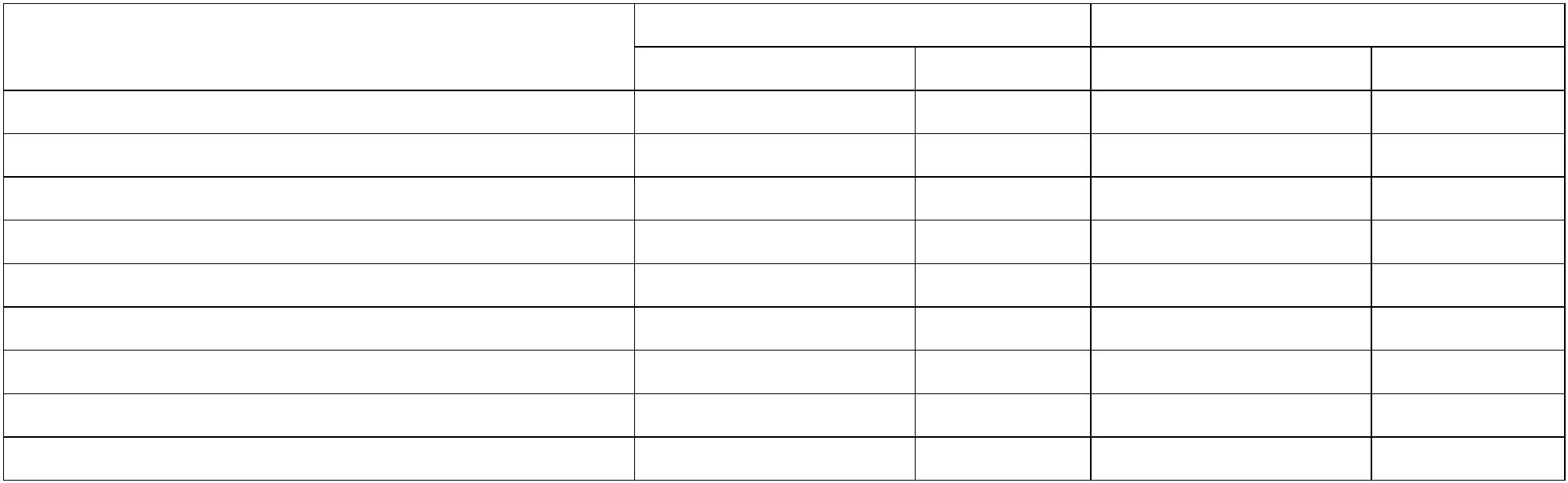


Table S12. The hazard ratio of low NME6 expression & early TNM stage group adjusted for age and sex in 5-year DFS and DSS

DFS

DSS

Variables

HR (95% CI)

P value

0.159

HR (95% CI)

1

P value

0.24

Protein expression & early TNM stage

High NME6 expression & early TNM stage

1

Low NME6 expression & early TNM stage

1.66 (0.82–3.36)

1.60 (0.73–3.55)

Age (years)

< 65

1

1

≥ 65

1.94 (1.40–2.68)

< 0.001

0.009

2.00 (1.40–2.84)

< 0.001

0.07

Sex

Female

Male

1

1

1.55 (1.12–2.16)

1.38 (0.97–1.95)

Abbreviations: NME6, nucleoside diphosphate kinase 6; DFS, disease-free survival; DSS, disease-specific survival; HR, hazard ratio; CI, confidence interval.


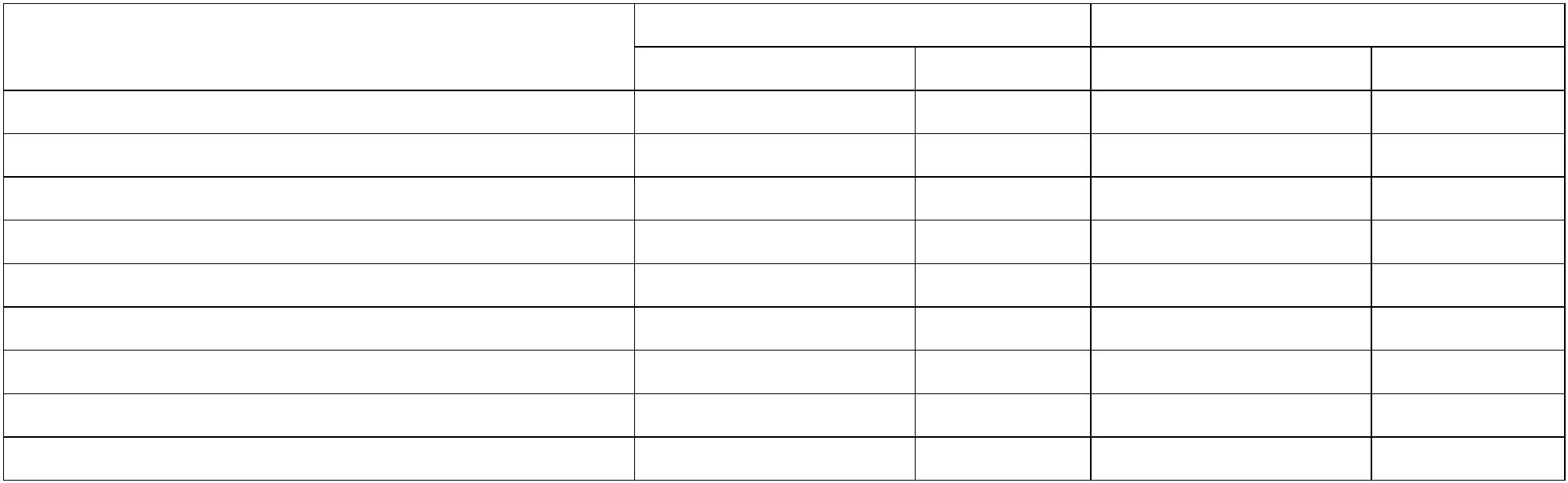


Table S13. The hazard ratio of low NME6 expression & late TNM stage group adjusted for age and sex in 5-year DFS and DSS

DFS

DSS

Variables

HR (95% CI)

P value

0.094

HR (95% CI)

1

P value

0.027

Protein expression & late TNM stage

High NME6 expression & late TNM stage

1

Low NME6 expression & late TNM stage

1.42 (0.94–2.15)

1.62 (1.06–2.48)

Age (years)

< 65

1

1

≥ 65

1.94 (1.40–2.68)

< 0.001

0.009

2.00 (1.40–2.84)

< 0.001

0.07

Sex

Female

Male

1

1

1.55 (1.12–2.16)

1.38 (0.97–1.95)

Abbreviations: NME6, nucleoside diphosphate kinase 6; DFS, disease-free survival; DSS, disease-specific survival; HR, hazard ratio; CI, confidence interval.


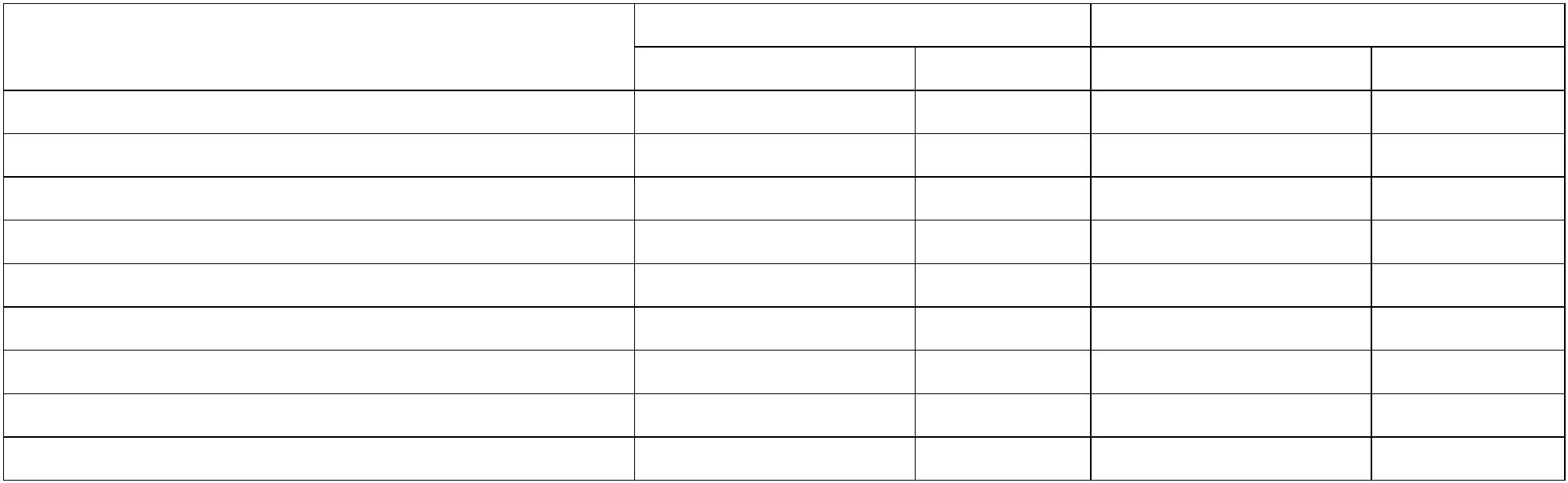


Fig. S1. Survival analysis according to the expression of five purine metabolic proteins in subgroups of GMC

cohort. Kaplan-Meier curves for DFS in wild-type TP53 (A), mutant TP53 (B), and MSS (C) subgroups were

depicted. Kaplan-Meier curves for DSS in wild-type TP53 (D), mutant TP53 (E), and MSS (C) subgroups were

depicted.


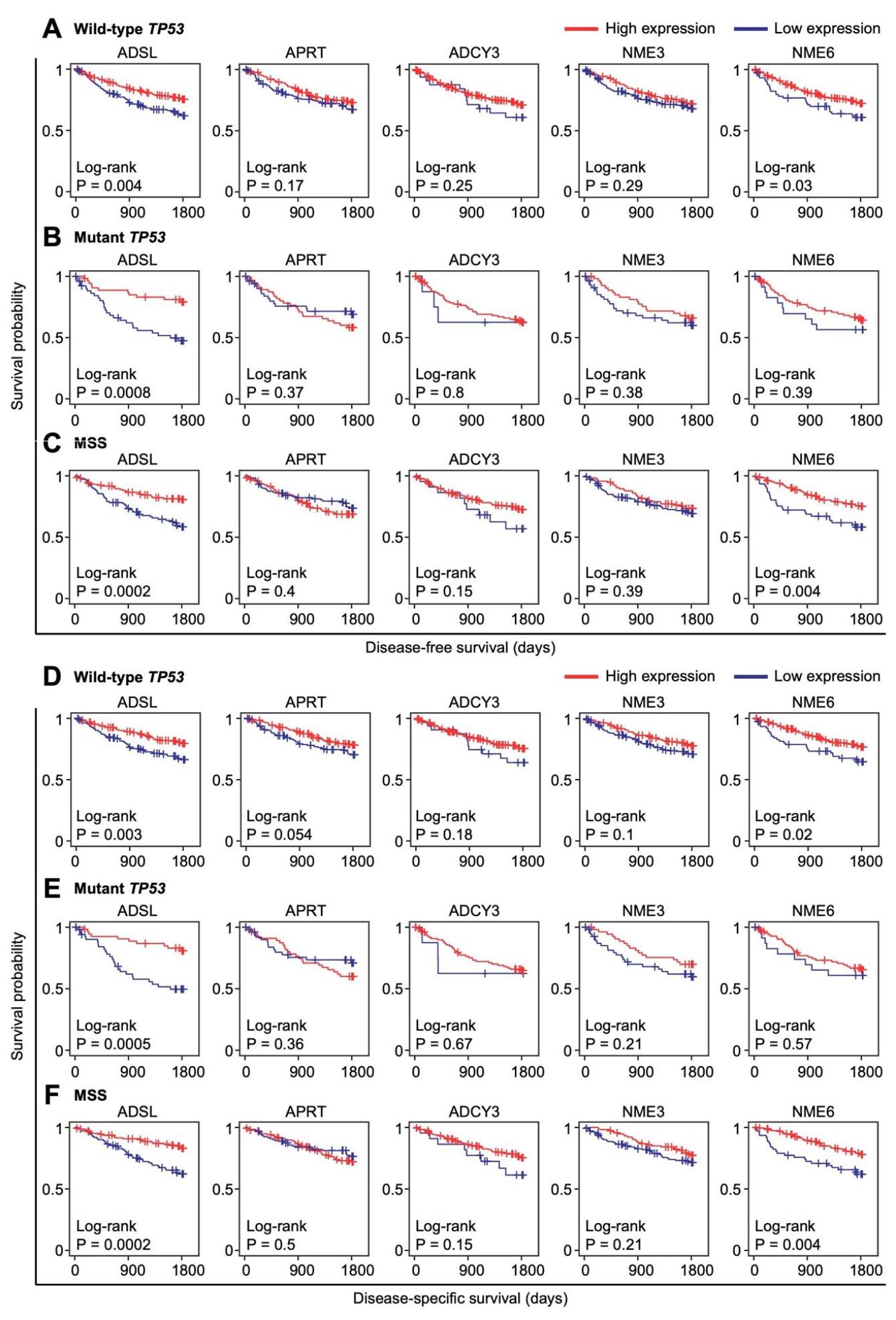


Fig. S2. Time-dependent ROC curves of five purine metabolism-related genes for predicting 1-, 3-, 5-year OS

outcome in GEO cohort. ROC curves in all patients, wild-type TP53, mutant TP53, and MSS subgroup were

depicted.


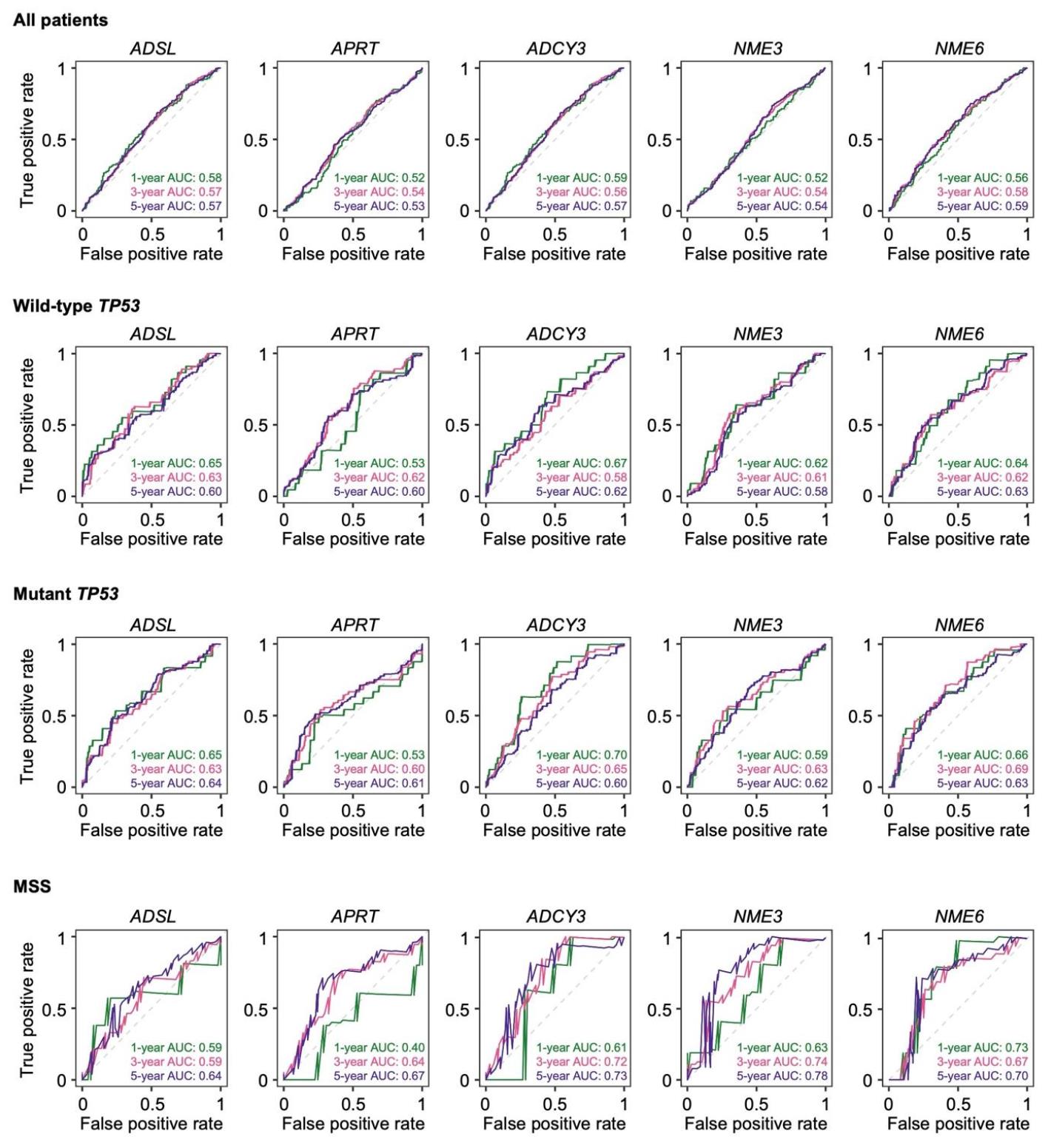


Fig. S3. Time-dependent ROC curves of five purine metabolic proteins for predicting 1-, 3-, and 5-year DFS

outcome in GMC cohort. ROC curves in all patients, wild-type TP53, mutant TP53, and MSS subgroup were

depicted.


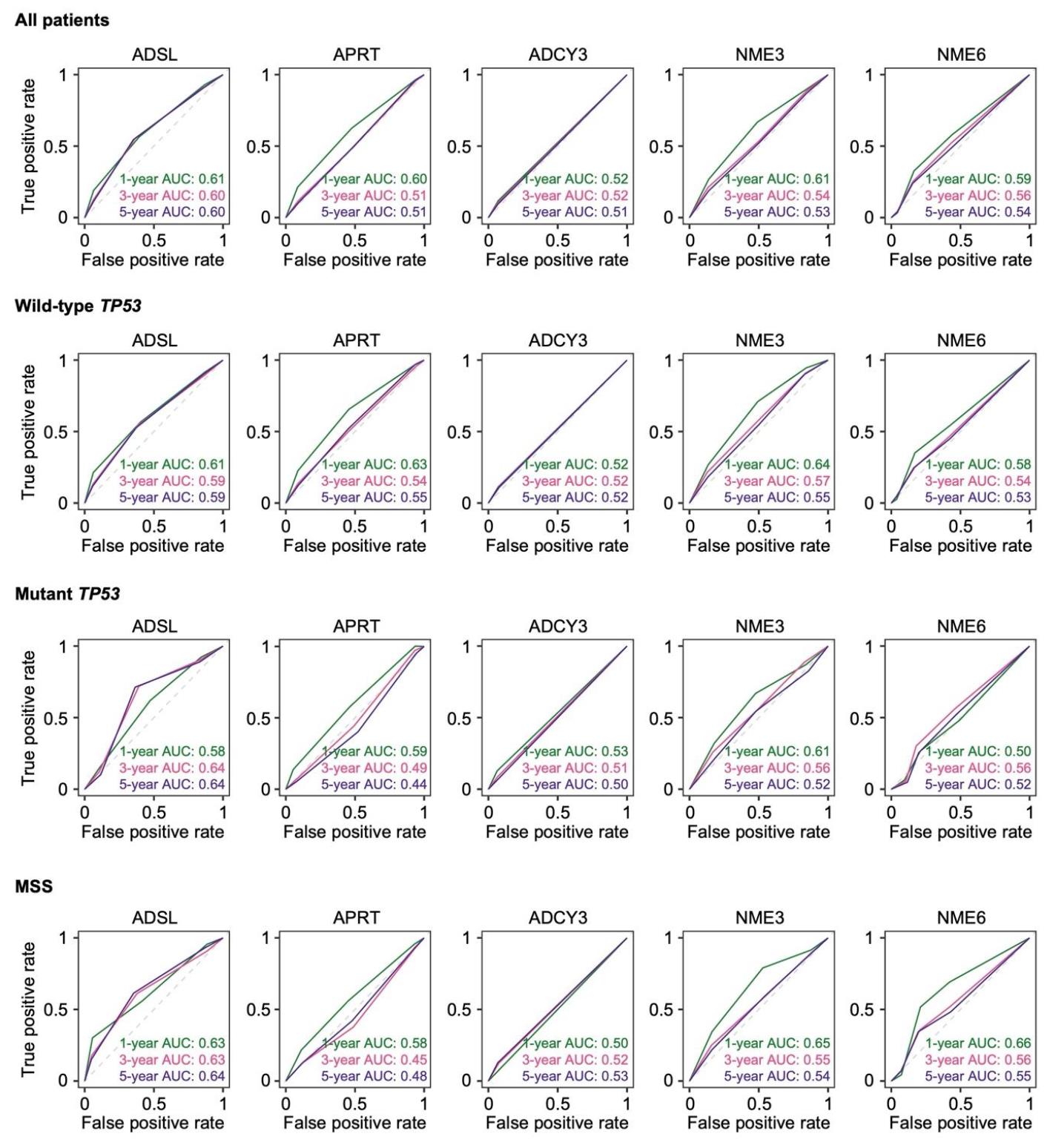


Fig. S4. Time-dependent ROC curves of five purine metabolic proteins for predicting 1-, 3-, and 5-year DSS

outcome in GMC cohort. ROC curves in all patients, wild-type TP53, mutant TP53, and MSS subgroup were

depicted.


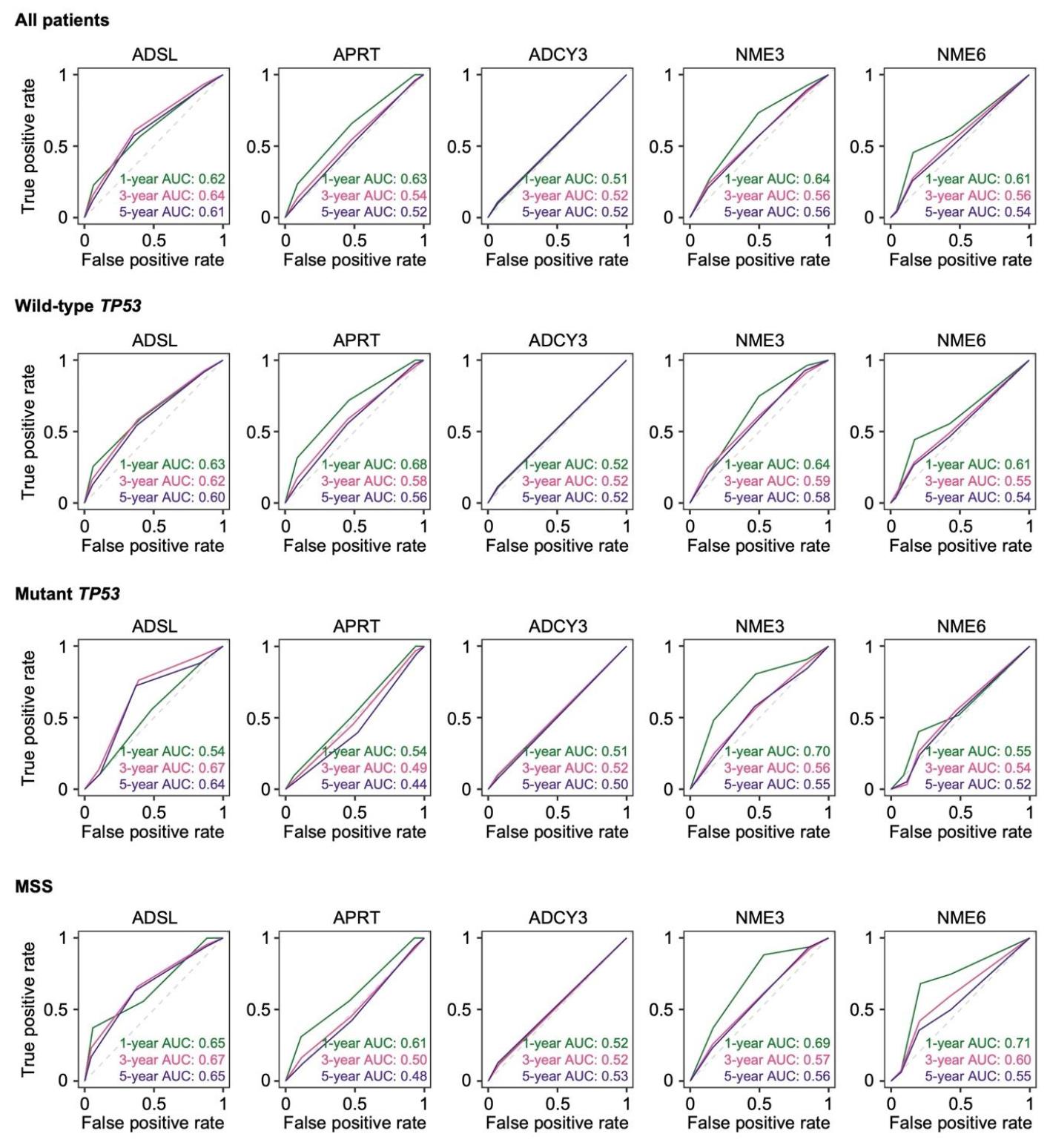

Supplement: Supplementary file 1 — Fig. S1. Survival analysis according to the expression of five purine metabolic proteins in subgroups of GMC cohort. Fig. S2. Time‐dependent ROC curves of five purine metabolism‐related genes for predicting 1‐, 3‐, 5‐year OS outcome in GEO cohort. Fig. S3. Time‐dependent ROC curves of five purine metabolic proteins for predicting 1‐, 3‐, and 5‐year DFS outcome in GMC cohort. Fig. S4. Time‐dependent ROC curves of five purine metabolic proteins for predicting 1‐, 3‐, and 5‐year DSS outcome in GMC cohort. Table S1. Summary of the GEO cohort. Table S2. Cutoff values of five purine metabolism‐related genes to classify into the low and high expressing group in all patients of GEO cohort. Table S3. Cutoff values of five purine metabolism‐related genes to classify into the low and high expressing group in subgroups of GEO cohort. Table S4. The hazard ratio of low ADSL expression and early TNM stage group adjusted for age and sex in 5‐year DFS and DSS. Table S5. The hazard ratio of low ADSL expression and late TNM stage group adjusted for age and sex in 5‐year DFS and DSS. Table S6. The hazard ratio of low APRT expression and early TNM stage group adjusted for age and sex in 5‐year DFS and DSS. Table S7. The hazard ratio of low APRT expression and late TNM stage group adjusted for age and sex in 5‐year DFS and DSS. Table S8. The hazard ratio of low ADCY3 expression and early TNM stage group adjusted for age and sex in 5‐year DFS and DSS. Table S9. The hazard ratio of low ADCY3 expression and late TNM stage group adjusted for age and sex in 5‐year DFS and DSS. Table S10. The hazard ratio of low NME3 expression and early TNM stage group adjusted for age and sex in 5‐year DFS and DSS. Table S11. The hazard ratio of low NME3 expression and late TNM stage group adjusted for age and sex in 5‐year DFS and DSS. Table S12. The hazard ratio of low NME6 expression and early TNM stage group adjusted for age and sex in 5‐year DFS and DSS. Table S13. The hazard ratio of low NME6 expressio [file MOL2-19-2310-s001.docx]
